# Preparation and Research Progress of Polymer-Based Anion Exchange Chromatography Stationary Phases

**DOI:** 10.3390/polym18030389

**Published:** 2026-01-31

**Authors:** Haolin Liu, Jingwei Xu, Yifan Shen, Shi Cheng, Yangyang Sun, Chendong Shuang, Aimin Li

**Affiliations:** 1 State Key Laboratory of Water Pollution Control and Green Resource Recycling, School of the Environment, Nanjing University, Nanjing 210023, China; liuhaolin@smail.nju.edu.cn (H.L.); 502024250075@smail.nju.edu.cn (J.X.); 18895667933@163.com (Y.S.); 2 Jiangsu Key Laboratory of E-Waste Recycling, School of Resources and Environmental Engineering, Jiangsu University of Technology, Changzhou 213001, China; shicheng@jsut.edu.cn; 3 Qingdao Shine Chromatograph Technology Company Limited, Qingdao 266101, China; sunyangyang@quenda.cn

**Keywords:** ion chromatography, stationary phase, polymer, functionalization

## Abstract

Ion chromatography (IC) serves as a pivotal technique in trace ion analysis, and the separation performance of IC is largely determined by the properties of stationary phases. In contrast to silica-based matrices, polymer-based stationary phases have garnered significant interest owing to their outstanding pH stability and mechanical robustness. However, unmodified polymer matrices usually lack necessary ion exchange functions and selectivity; therefore, precise functional modification is the key to improving their chromatographic separation performance. This paper provides a systematic overview of recent advances in the synthesis and functional modification of polymer-based anion exchange chromatography stationary phases over the past few years. Firstly, the types and characteristics of polymer matrices commonly used for functional modification are summarized; secondly, the origin and improvement of common synthesis methods such as microporous membrane emulsification, droplet microfluidics, suspension polymerization, emulsion polymerization, soap-free emulsion polymerization, precipitation polymerization, dispersion polymerization, and seed swelling are introduced according to the molding methods of polymer matrices; furthermore, the principles, characteristics, and development status of mainstream functionalization strategies, including chemical derivatization, surface grafting, latex agglomeration, and hyperbranching, are emphasized. Finally, the existing challenges and prospective development trends in this field are discussed and outlooked, with the purpose of offering insights for the targeted design and practical application of high-performance polymer-based anion exchange chromatography stationary phases.

## 1. Introduction

Ion chromatography (IC) is the core technology for analyzing anions in environmental samples [1], and its development has always been closely linked to the needs of environmental monitoring [2]. From the early routine detection of common anions and cations to the current precise analysis of trace pollutants in complex matrices by coupling with mass spectrometry [3,4,5,6], each significant innovation in stationary phase performance has promoted the progress of IC technology [7,8].

The stationary phase is recognized as the “heart” of IC, and its physical characteristics (including particle size, pore diameter, and mechanical robustness) and chemical attributes (such as matrix reactivity and functional moieties) collectively govern the separation efficiency, selectivity, and stability of the chromatographic column. The matrix serves as the support for functional groups, and its inherent properties directly constrain the overall performance of the stationary phase. The silica-based matrix widely used in the early stage has a narrow pH tolerance range (2~8), so it faces many challenges in ion chromatography sample analysis. Therefore, silica-based anion exchange stationary phases cannot be used with mobile phases commonly used in suppressed IC mode, and the sensitivity of non-suppressed ion chromatography is low, making silica-based stationary phases only applicable to high-concentration samples. To meet the growing demand for high selectivity, high sensitivity, and high-throughput analysis in the environmental field, polymer-based materials have gradually emerged [9]. Polymer matrices are typically fabricated via the cross-linking polymerization of organic monomers, including styrene, divinylbenzene, and methyl methacrylate. Compared with traditional silica-based matrices, polymer matrices demonstrate a broader pH applicability range, thus rendering them extensively employed as the matrix for ion chromatography stationary phases. Beyond silica and polymer microspheres, a variety of emerging materials, including metal oxides, carbon quantum dots, covalent organic frameworks (COFs), and metal–organic frameworks (MOFs), are also available for selection [10,11,12,13]. Besides matrix selection, the functional moieties present on the matrix surface exert a crucial influence on the overall performance of ionic adsorbents and ion chromatographic stationary phases. Specifically, for the adsorption and separation of cationic species, the surfaces of the employed adsorbents and chromatographic packings are primarily modified with negatively charged functional groups—for instance, carboxylic acid and sulfonic acid—while, for the adsorption and separation of anionic substances, the charged functional groups on the material surface are mainly protonated amino functional groups and quaternized amino functional groups. In 2016, researchers [14] reviewed the preparation and functionalization of polymer-based ion chromatography stationary phases, but nearly 10 years have passed, and many developments related to hyperbranching and surface grafting have not been included; in 2021, researchers [15] reviewed the preparation of ion chromatography stationary phases, but focused on the introduction of all stationary phase matrices and chromatographic applications, so the introduction to the synthesis of stationary phase matrices, especially polymer matrices, is limited. Consequently, this review is intended to comprehensively recapitulate the recent advances regarding the synthesis of matrices and the surface functionalization of polymer-based anion exchange chromatography stationary phases over the past few years.

## 2. Synthesis of Polymer Matrices

Organic polymer matrices are the mainstream packing materials for commercial ion chromatography columns and are widely used. Commercial ion chromatography columns from companies such as Thermo Fisher Scientific (Waltham, MA, USA), Metrohm AG (Herisau, Switzerland), and Tosoh Corporation (Tokyo, Japan) mostly use such packing materials. Their core advantage lies in the wide pH tolerance range: for example, polymethacrylate and polyvinyl alcohol microspheres can be stably used at pH 2–12 [16,17], while polystyrene–divinylbenzene (PS-DVB) and ethylvinylbenzene–divinylbenzene (EVB-DVB) can withstand the entire pH spectrum from 0 to 14, a characteristic that enables the use of ultra-high pH eluents—for instance, hydroxide solutions. Furthermore, the compatibility of organic polymer matrices with organic solvents permits the application of strong acids or bases as eluents, which facilitates the conversion of neutral and poorly dissociable compounds (e.g., weak acids and organic amines) into ionizable species, thus broadening the analytical applicability of ion exchange chromatography.

### 2.1. Types of Polymer Matrices

Organic polymer matrices are the mainstream packing materials for commercial ion chromatography columns. By precisely regulating the monomer ratio and polymerization conditions, the physical structure of the stationary phase can be directionally controlled; common organic polymer matrices include the styrene–divinylbenzene polymer [18,19], ethylvinylbenzene–divinylbenzene copolymer [20,21], methyl methacrylate polymer [22], and polyvinyl alcohol polymer [23], etc.

The inherent characteristics of organic polymer matrices still bring two core challenges. Firstly, the mass transfer efficiency bottleneck: many polymer matrices (especially high-cross-linking-degree resins) have limited porosity and specific surface area (SSA) and a poor pore size distribution, resulting in a hindered mass transfer and an impaired diffusion of analytes within the stationary phase, which becomes a key limiting factor for improving chromatographic column efficiency (especially for macromolecules or ions with slow diffusion).

To address these challenges, research strategies are evolving towards microstructure design and functional composites. On the one hand, through innovations in synthetic methodologies such as microfluidics and controlled seed swelling, the polymerization process is precisely regulated to construct polymer skeletons with hierarchical pore structures, thereby increasing the SSA, improving mass transfer efficiency, and reducing column pressure.

This chapter will focus on discussing some of the most representative matrix structure types in modern packed IC columns.

#### 2.1.1. Polystyrene–Divinylbenzene Matrix

Monodisperse cross-linked polystyrene–divinylbenzene (PS-DVB) microspheres have emerged as among the most extensively adopted matrix substrates for ion chromatography packings on account of their sizeable SSA, excellent chemical stability, broad pH applicability range, and tunable pore size [18,19]. The skeleton of PS-DVB microspheres is mainly composed of three parts: styrene monomers form the main body of the matrix; divinylbenzene serves as a cross-linking agent, and its content determines the cross-linking degree of the microspheres, thereby regulating the pore structure and water content of the resin; and functional groups bonded to the skeleton provide active sites for the interaction with target analytes.

The cross-linking degree stands as a critical parameter for modulating the performance of PS-DVB resin, exerting a direct impact on its pore size distribution, mechanical strength, swelling property, and pressure resistance in organic solvents. Increasing the cross-linking degree helps to enhance the hardness, thermal stability, and chemical corrosion resistance of the resin, but an excessively high cross-linking degree may lead to an increased brittleness of the resin, decreased swelling degree, reduced ion exchange capacity, and hindered column efficiency. Research findings have demonstrated that anion exchange chromatography stationary phases derived from PS-DVB and EVB-DVB matrices exhibit no shrinkage or swelling behavior in 100% organic solvents when their cross-linking degree attains 55% [24]. Therefore, it is necessary to weigh the pros and cons in the preparation of chromatographic packings and select the optimal cross-linking degree.

These research progresses indicate that, through the whole-process precise regulation of the PS-DVB matrix during the synthesis stage, its physical behaviors such as SSA, particle size, and monodispersity can be changed, endowing it with better chromatographic performance and a wider applicability in the separation of complex environmental samples [25].

#### 2.1.2. Polyacrylate-Based Matrix

The polymethyl methacrylate (PMMA) polymer matrix is a relatively new development in the field of ion chromatography stationary phases [26,27]. This type of matrix is usually copolymerized with DVB, ethylene glycol dimethacrylate, etc., as cross-linking agents and has a good stability. Its notable merit lies in the fact that it exhibits a greater hydrophilicity compared to PS-DVB and EVB-DVB, and is fully compatible with all HPLC solvents—in other words, high-concentration organic solvents (such as methanol, acetonitrile, etc.) can be incorporated into the mobile phase, thus enabling the flexible modulation of selectivity. This feature proves especially advantageous for separating anionic pollutants with substantial hydrophobic disparities in complex aquatic environments.

The PMMA matrix has a high chemical reactivity—the epoxy groups in its molecules have a strong reactivity and are easy to modify to introduce various functional groups. The hydrophilicity of the PMMA matrix can be strengthened through the hydrolysis of epoxy groups using dilute sulfuric acid to generate hydroxyl groups. In comparison with the PS-DVB matrix, it offers the benefit that ligands can be immobilized on microspheres via a one-step reaction, but its disadvantage is that its suitable pH range is narrower than that of PS-DVB microspheres, and ester bonds limit its application in suppressed ion chromatography analysis under extreme pH conditions [28].

### 2.2. Preparation of Polymer Matrices

During the late 1960s, monodisperse polystyrene (PS) microspheres with a particle size range of 2~30 μm were fabricated in outer space via emulsion polymerization by Vanderhoff et al. [29]; nevertheless, the prohibitive cost of these microspheres posed an obstacle to large-scale industrial application. Subsequently, the preparation technology of monodisperse polymer microspheres has emerged as a key research focus in the field of polymer materials and related interdisciplinary areas. At present, researchers aim to prepare polymer microspheres with a high monodispersity, easy surface modification, and controllable morphology and structure by exploring the influence of various preparation parameters during the polymerization process on chemical and physical properties such as the particle size, morphology, and pore size of microspheres. Following decades of application and development, researchers have carried out more in-depth studies on the preparation procedures of monodisperse polymer microspheres. Gokmen et al. [30] have comprehensively reviewed the synthesis methods of porous polymer microspheres, but this paper mainly divides them into two categories, processing molding methods and polymerization molding methods, according to the molding methods of the microspheres.

#### 2.2.1. Processing Molding Methods

Processing-based forming techniques denote initially synthesizing oligomeric substrates, followed by the formation of polymer microspheres via subsequent processing steps. Such subsequent processing typically employs physical approaches, including external pressure and intermolecular interactions, for microsphere fabrication. The specific procedure of most processing molding methods involves first fabricating oil-in-water (O/W), water-in-oil (W/O), O/W/O, or W/O/W emulsions and then adopting a suitable curing method based on the characteristics of the polymer matrix to convert the droplets into microspheres. In terms of the functionalization of microspheres, polymer functional microspheres can be obtained by adsorbing molecules or particles with functional groups during spheroidization, or by performing functionalization simultaneously during processing. Processing molding methods have great advantages for preparing microspheres, adopted in industries such as drug delivery, biological analysis, and food, since the oligomers synthesized can use natural polymer raw materials, such as starch, albumin, cellulose, etc. The monodisperse polymer microspheres fabricated through this approach possess numerous merits including biodegradability, high purity, excellent biocompatibility, non-toxicity, and safety [31]. Currently, the processing-based forming techniques for microspheres primarily encompass phase separation, emulsification solvent evaporation, droplet microfluidic technology, and microporous membrane emulsification. Among these, relatively widely employed techniques for the preparation of polymer-based ion chromatographic stationary phases in processing-based molding methods are droplet microfluidic technology and microporous membrane emulsification. This chapter will only introduce these two microengineering emulsification technology methods in detail.

##### Microporous Membrane Emulsification Method

**Figure 1:**
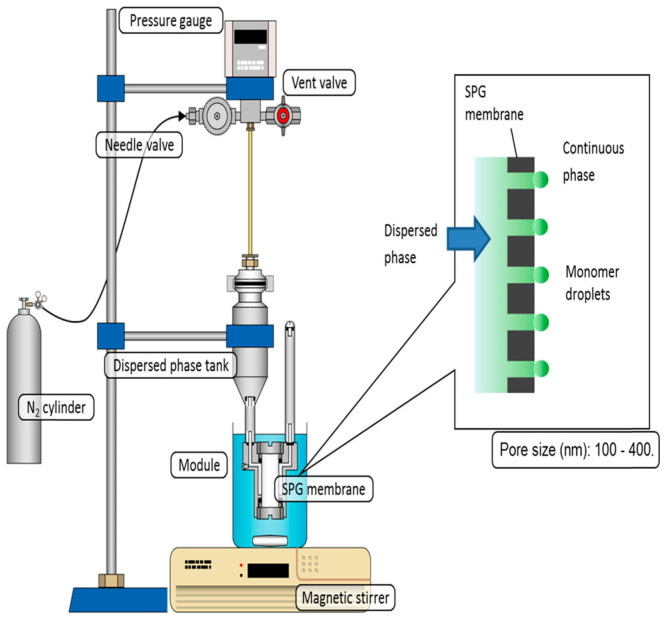
Schematic diagram of SPG membrane emulsification method. Reprinted with permission of American Chemical Society from [34].

The microporous membrane emulsification technique emerged during the late 1980s. Nakashima et al. [32] manufactured a porous glass membrane of CaO-Al_2_O_3_-B_2_O_3_-SiO_2_, called the Shirasu Porous Glass (SPG) membrane, and used it in preparing monodisperse polymer microspheres. Now, this technical method is also called glass membrane emulsification method. The SPG membrane is a selectively permeable membrane featuring a uniform microporous structure, and artificial selection allows for the control of its pore size [33]. The operational schematic diagram of this technology is presented in Figure 1 [34]. The operational process entails that the emulsion, composed of solvents and monomers, is dispersed into continuous phase a, after which it is forced to penetrate the pores of the SPG membrane under applied pressure. The emulsion undergoes a gradual dispersion into the immiscible phase b as droplets, and a favorable sphericity is achieved after the solvent in phase b evaporates, or in conjunction with suspension polymerization. Due to the high uniformity of the micropores of the SPG membrane, the emulsion will form uniform droplets when passing through its pores, resulting in polymer microspheres with good consistency. Hao et al. [35] used the microporous membrane emulsification method to emulsify the organic phase composed of toluene, DVB, n-hexane, and an initiator, and then prepared monodisperse macroporous polydivinylbenzene (PDVB) microspheres through suspension polymerization. It was found in the experiment that the dosage and type of porogens exert a notable influence on the SSA of the microspheres. Sugiura et al. [36] emulsified DVB dissolved with benzoyl peroxide using the microchannel emulsification method, and then obtained PDVB microspheres through suspension polymerization. By adjusting the microporous membrane’s pore size, monodisperse PDVB microspheres with an average particle size (APS) of 69.0 μm and a particle size coefficient of variation of 4.1%, as well as those with an APS of 86.7 μm and a particle size coefficient of variation of 4.8%, were successfully fabricated in sequence. Miki et al. [37] employed trimethylolpropane trimethacrylate and DVB as the monomers, using toluene as the porogen. An O/W emulsion was initially prepared via the SPG membrane emulsification technique, followed by suspension polymerization, to produce monodisperse porous polymer microspheres—these microspheres had a particle size range of 50~60 μm and a coefficient of variation (CV) of 9.5%. Nida Nauman et al. [34] coupled the SPG membrane emulsification method with miniemulsion polymerization to fabricate monodisperse PMMA microspheres possessing a particle size spanning 250~1600 nm.

##### Droplet Microfluidic Technology

**Figure 2:**
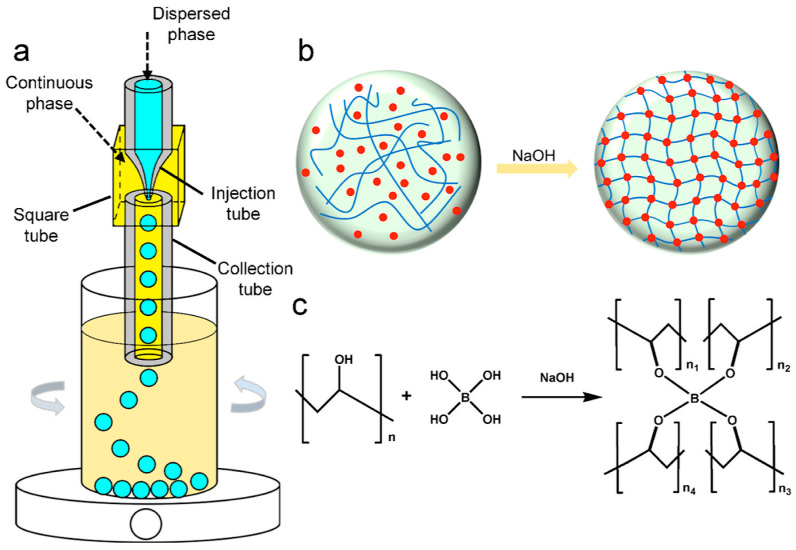
Schematic diagram of the microfluidic device and cross-linking principle pertaining to the fabrication of monodisperse poly(vinyl alcohol) (PVA) microspheres. (**a**) Microfluidic droplet generation: monodisperse PVA droplets. (**b**) NaOH triggering: in-droplet cross-linking and solidification. (**c**) Borate ester bridging: formation of a 3D network. Reprinted with permission of American Chemical Society from [39].

Microfluidic technology is defined as a system that designs and employs microchannels with a dimensional scope of tens to hundreds of micrometers to handle and manipulate microfluids. It constitutes an interdisciplinary integrated technology encompassing physics, chemistry, fluid mechanics, microelectronics, and other fields [38]. Droplet microfluidic technology serves as a key branch of microfluidic technology, representing a technique for the precise handling of small liquid volumes. Its core principle is to separate the continuous phase into a multitude of monodisperse droplets through the interaction of shear force and surface tension in the microchannel, or using other external forces, and finally solidify to obtain monodisperse polymer microspheres (Figure 2) [39]. At present, the droplets generated using droplet microfluidic technology are mainly divided into passive as well as active types [40].

Designing the geometric configuration of microchannels and regulating fluid flow rates are the primary means of achieving passive droplet generation, which serves to disperse the polymer matrix. Driven by viscous force, fluid shear force, and interfacial tension, droplets are formed by the dispersed phase at the flow field junction. Currently, the widely employed droplet microfluidic chip structures mainly include Co-flow, Flow focusing, and T-junctions. Although the chip structure is a key factor affecting droplet size and morphology, parameters including physical properties, fluid flow rate, and interfacial tension also have an influence on the droplet formation process [41]. For the purpose of detailing the technological progress in practical droplet generation, droplet generation methods were first categorized into modern and classical technologies by Shobhit Das et al., and were further classified into active and passive approaches based on their operating mechanisms [42].

Compared with traditional technologies, droplet microfluidic technology has many advantages, including a fast reaction speed, high controllability, and no pollution [43,44]. In recent years, there have been many studies on preparing monodisperse polymer microspheres using microfluidic technology. The size of droplets can be accurately regulated using microfluidic technology, so monodisperse microspheres with controllable internal architectures can be prepared using droplets as templates. However, the microspheres fabricated with this technology are primarily applied in chemical and biopharmaceutical fields such as pharmaceutical engineering, drug delivery, and tissue engineering, yet studies on their application in the field of chromatographic stationary phases remain scarce. Zhang et al. [45] prepared four types of monodisperse polymer microspheres (polyvinyl chloride (PVC), polylactic acid (PLA), polystyrene (PS), and polyvinylidene fluoride–hexafluoropropylene (PVDF-HFP)) using microfluidic technology. The particle sizes of the polymer microspheres are capable of being readily adjusted by modifying process parameters, and the fabricated microspheres feature a narrow size distribution and exhibit higher efficiency compared with other technologies like emulsion polymerization. Porous poly(lactic-co-glycolic acid) (PLGA) particles, namely highly open porous microspheres (HOPMs), were fabricated via microfluidic technology by Kankala et al. [46]. They adopted Minitab software version 17 full-factorial experimental design to explore the effects of the water-oil ratio, polymer concentration, and porogen (gelatin) concentration on pore size—all these formulation parameters were confirmed to exert significant impacts (*p* < 0.05) on the pore diameter of the HOPMs. Chen et al. [47] devised an efficient approach integrating microfluidic technology and the interface instability principle to fabricate monodisperse PLGA-PEG/PLGA microspheres with customized surface morphologies. Through tuning the mass ratio between Poly(Lactic-Co-Glycolic Acid)-Polyethylene Glycol (PLGA-PEG) and PLGA, stabilizer dosage, and PLGA type, porous microspheres with distinctive folded morphologies—including “lace-like”, “fish tail-like”, and “sponge-like” structures—were produced.

Droplet microfluidic technology is capable of yielding monodisperse polymer microspheres, and the entire system exhibits excellent controllability along with a promising application potential [48]. Nevertheless, the high manufacturing cost and technical barrier of this technology result in elevated microsphere fabrication costs, and its further industrial implementation still awaits in-depth exploration.

#### 2.2.2. Polymerization Molding Methods

Key strategies for fabricating polymer microspheres with monodisperse features via polymerization-based forming techniques encompass suspension, emulsion, precipitation, soap-free emulsion, dispersion, and seed polymerization. Microspheres fabricated via these techniques typically exhibit distinct particle sizes. Table 1 summarizes the reaction features, advantages, and drawbacks of these methods.

##### Suspension Polymerization

Suspension polymerization refers to a technique where water serves as the medium—monomers are dispersed into small droplets via mechanical agitation, so as to disperse and suspend in a stabilizer-containing aqueous phase, followed by the initiation of polymerization [49]. According to the water solubility of the polymer, suspension polymerization is divided into homogeneous and heterogeneous reactions. Usually, a dispersant needs to be introduced to reduce the surface tension of the reaction system and stably disperse the monomers into droplets. The dispersants used are mainly water-soluble polymer dispersants, such as polyvinylpyrrolidone, polyvinyl alcohol, gelatin, and hydroxymethyl cellulose. Azo compounds (e.g., azobisisobutyronitrile) or organic peroxides (e.g., benzoyl peroxide) are commonly employed as initiators, with the polymerization temperature ranging from 50 to 100 °C. Porogens may include good solvents (such as toluene, benzene, dichloromethane), poor solvents (e.g., n-hexane), linear polymers (e.g., polymethyl methacrylate, polystyrene), or mixed porogens—all of which can be utilized to regulate the pore structure of the polymer.

Conventional suspension polymerization can be adopted to fabricate polymer microspheres with a particle size of 2~2000 μm [50], which are low in price, safe to produce, and easy to separate into products, while their reaction system has a low viscosity, so the heat can be simply taken away using cooling water, and the temperature is uncomplicated to control. In suspension polymerization, the stirring speed of the mechanical stirrer is a key factor affecting the particle size distribution (PSD) [51]. Additionally, the volume proportion of the organic phase to the aqueous phase, their respective viscosities, and stabilizer concentration exert an influence on the microspheres’ particle size. An elevated agitation speed leads to a decrease in the particle size of the microspheres. During the entire reaction process, continuous mechanical stirring at a constant speed is required to ensure the sufficient dispersion of the droplets. However, in actual operation, due to the formation of droplets still being controlled by chaotic stirring, the collision and fragmentation of droplets often occur during the entire reaction process. The particles obtained only through suspension polymerization are almost always polydisperse and need to be sieved as the matrix of IC stationary phases [30]. This constitutes the primary drawback of this technique. Thus, in the preparation of monodisperse polymer microspheres, suspension polymerization is predominantly combined with droplets fabricated via processing-based forming methods.

During the preparation of porous PS-DVB microspheres via suspension polymerization, the dosage and type of porogens serve as the primary factors governing the pore structure of PS-DVB microspheres [52]. The dosage of DVB also exerts a significant impact on their pore structure—an elevation in its dosage will enhance the pore size and reduce the particle size [53,54]. Erbay et al. [55] prepared PS-DVB microspheres through suspension polymerization. Under the same other conditions, toluene, toluene/cyclohexanol (volume ratio 75/25), and cyclohexanol were used as porogens, respectively. The number of pores of the microspheres in the range of 10~100 nm increased in turn; the average pore size increased, and a large number of pores of 100~1000 nm appeared when cyclohexanol was used as the porogen. Liu et al. [56] employed linear polypropylene dissolved in toluene as the porogen (with a mass fraction of 6.0%). They found that, with the total volume of DVB and styrene kept constant, as the volume ratio of DVB to styrene rose from 1:3 to 3:0, the SSA of the microspheres escalated from 91 m^2^/g to 608 m^2^/g, while the average pore size determined via mercury porosimetry declined from 37.3 nm to 16.5 nm. With the dosage of DVB held fixed, as the volume ratio of porogen to DVB increased from 2:3 to 4:3, the SSA of the resulting microspheres climbed from 106 m^2^/g to 652 m^2^/g, the average pore size detected through mercury porosimetry increased from 11.8 nm to 22.7 nm, and the pore volume expanded from 0.31 cm^3^/g to 0.90 cm^3^/g.

##### Emulsion Polymerization

Emulsion polymerization is an important technology for preparing polymer microspheres [57] and a relatively traditional method for preparing polymer microspheres, with a development history of more than 70 years. In the 1940s, Harkins et al. [58] proposed the micellar nucleation theory for the mechanism of emulsion polymerization, analyzed the three stages of emulsion polymerization, and established the classic emulsion polymerization theory. Currently, it is generally accepted that the emulsion polymerization of hydrophobic monomers (e.g., styrene) can be elucidated using this model; another theoretical model illustrating the mechanism of emulsion polymerization is the homogeneous nucleation theory [59], which is suitable for the polymerization of hydrophilic monomers (e.g., methyl methacrylate). Emulsion polymerization exhibits the following advantages: (1) Since the polymerization system maintains in a continuous phase state throughout the reaction process, the reaction heat produced via free radical polymerization can be transferred and dissipated through the aqueous phase in a simple way. (2) The polymerization rate of emulsion polymerization is generally much more elevated than that of bulk polymerization, while the molecular weight (MW) of the resulting polymers is also notably higher than that of products from bulk polymerization. (3) Water serves as the reaction medium for both the polymerization process and the resultant product latex, which imposes modest requirements on the reaction equipment; additionally, the reaction process entails fewer safety hazards and environmental pollution issues [60]. However, emulsion polymerization itself also has certain disadvantages. On the one hand, emulsion polymerization can only prepare monodisperse microspheres with a particle size below 1 μm in most cases, which severely limits its application; on the other hand, owing to the requirement of emulsifiers in the polymerization system, the resulting polymerization product inherently retains residual emulsifiers that are challenging to remove thoroughly. Such drawbacks restrict the further large-scale implementation of emulsion polymerization. Table 2 summarizes several commonly used emulsifiers.

##### Soap-Free Emulsion Polymerization

Soap-free emulsion polymerization is a new technology engineered on the basis of the classic theory of emulsion polymerization. The so-called soap-free emulsion technology refers to the emulsion polymerization process that does not use or only uses a smattering of emulsifiers [61]. Previous researchers included soap-free emulsion polymerization in the same introduction as emulsion polymerization in reviews introducing ion chromatography stationary phases [15]. Indeed, both are polymerization reactions carried out in an aqueous dispersion medium; the monomers are hydrophobic, with very little solubility in water, and the final products are stable latex systems in which polymers are dispersed in water in the form of submicron colloidal particles. However, considering that the formation mechanism and stability conditions of latex particles are completely different from those of classic emulsion polymerization [62,63], this method is independently introduced in this paper. Traditional emulsion polymerization relies on externally added small-molecule emulsifier molecules physically adsorbed on the particle surface to provide electrostatic repulsion or steric hindrance to prevent particle aggregation, while soap-free emulsion polymerization chemically bonds stabilizing groups (such as sulfonic acid groups, carboxylic acid groups) to the ends or surfaces of polymer chains through chemical reactions. For example, using persulfate initiators, their fragments (—SO_4_^−^) will remain on the particle surface as hydrophilic ends and copolymerize a small amount of hydrophilic monomers (such as methacrylic acid, acrylic acid), whose hydrophilic segments will enrich at the particle–water interface. Since there are almost no emulsifiers in the soap-free emulsion polymerization system, the colloidal stability of latex particles in the polymerization system is mainly achieved by combining ionic groups and hydrophilic groups on the polymer molecular chains or their end groups. These groups are usually introduced into the reaction system using the following three reactants: (1) introducing ionic groups using initiators, such as potassium persulfate; (2) hydrophilic comonomers; and (3) ionic comonomers.

Oliver J Deane et al. [64] used the poly(2-(N-acryloyloxy)ethylpyrrolidone) (PNAEP) macromolecular RAFT reagent of trithiocarbonate as a stabilizer to synthesize polymer microspheres such as styrene and butyl acrylate (n-BA) through RAFT soap-free emulsion polymerization. The results showed that the APS of the polymer microspheres was 99 nm, the particle dispersity PSD = 0.08, and it had good dispersibility. Zhu et al. [65] developed a one-step, soap-free emulsion polymerization route to prepare polystyrene nanoparticles with a good dispersity and tunable sizes of about 30–300 nm, without the need for large amounts of surfactant. The key strategy is the introduction of an ionic comonomer, sodium p-styrenesulfonate (NaSS), which copolymerizes with styrene during the reaction and imparts additional surface charges to the nascent particles, thereby enhancing colloidal stability and suppressing early-stage particle coagulation. The authors systematically investigated how monomer conversion, particle number, and particle size vary with NaSS content and polymerization time, and they also analyzed in detail the evolution of particle size distribution. The underlying mechanism can be summarized as a cooperation between the suppression of early irreversible aggregation and later-stage competitive growth coupled with a self-sharpening effect. This work offers a practically useful process and theoretical basis for producing small-sized, low-impurity polymer nanoparticles via soap-free emulsion polymerization.

Soap-free emulsion polymerization is an innovation and development of traditional emulsion polymerization, aiming to solve the problem of emulsifier residue and obtain polymer microspheres with a clearer structure and better performance. Given that little to no emulsifiers are involved in the polymerization process, the resultant polymerization products feature a high purity. Additionally, as the stability of latex particles is governed by surface functional groups, their structures are theoretically amenable to customized design. Consequently, the monodispersity of polymer microspheres fabricated via soap-free emulsion polymerization outperforms that of products prepared using conventional polymerization approaches. Table 3 lists the differences between traditional emulsion polymerization and soap-free emulsion polymerization. Nevertheless, the soap-free emulsion method still fails to surmount the drawback of the excessively small particle size of polymer microspheres. Additionally, it exhibits a sluggish polymerization rate, inferior emulsion stability, and poses challenges for industrialization. Meanwhile, studies on the nucleation mechanism of soap-free emulsion polymerization and the formation of latex particles still constitute a major challenge for researchers.

##### Precipitation Polymerization

**Figure 3:**
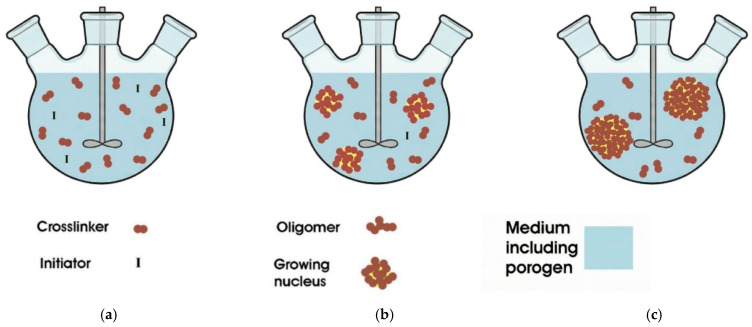
Flowchart of the precipitation aggregation method (in precipitation polymerization for porous particles, the process begins with a medium containing solely cross-linker and initiator molecules (**a**); radical polymerization then initiates, resulting in the formation of oligomers and primary nuclei (**b**); these nuclei subsequently grow by assimilating monomers and oligomers from the medium, with the nuclei being encompassed by a swollen oligomeric layer during this growth phase (**c**)).

In 1993, the Stover research group [66] prepared monodisperse PS-DVB polymer microspheres and other copolymer microspheres using acetonitrile as the reaction solvent, and proposed precipitation polymerization on this basis. It refers to the polymerization reaction of monomers in the reaction solvent to generate polymers that are insoluble in the solvent or whose polymer chains exceed the solubility of the solvent, thereby precipitating and separating from the solvent to finally obtain polymer microspheres [67], which still is a heterogeneous polymerization method [68]. Initially, the reaction medium mixed with monomers only contains a cross-linking agent and initiator molecules; then, in the nucleation stage, monomers first polymerize at the phase interface, and, as oligomers are generated and grow, they reach critical conditions for nucleation. Although oligomers are still soluble in the reaction medium, nuclei precipitate at this time to form a heterogeneous mixture; finally, in the nucleation and growth stage, after oligomers nucleate, monomers and cross-linking agents continue to interact on the surface of oligomers to form microspheres. Some double bonds of cross-linking agents are connected to microspheres, and the other double bonds will continue to attract monomers soluble in the reaction medium to make the microspheres grow further. Figure 3 is a flowchart of precipitation polymerization. Precipitation polymerization is typically employed for fabricating monodisperse highly cross-linked microspheres. During the fabrication process, the resultant microspheres are capable of maintaining structural independence via a self-stabilization mechanism, obviating the need for additional stabilizers [69], thus yielding products with a high purity. However, the monomer concentration in the system is low (2~5%), the reaction rate is slow, the yield is low, and a large amount of continuous phase solvent will cause environmental pollution. These factors restrict the widespread adoption of precipitation polymerization. Tan et al. [70] fabricated monodisperse PS-DVB (polystyrene–divinylbenzene) microspheres via precipitation polymerization, with acetic acid serving as the reaction medium. They examined the impacts of monomer concentration, initiator concentration, cross-linking agent dosage, temperature, and reaction time on the particle size and morphology of the microspheres. Under optimized conditions, monodisperse PS-DVB microspheres with a particle size spanning from 1.6 to 1.8 μm were obtained. Tugrul et al. [71] synthesized polymer microspheres with a narrow PSD and a micrometer-range APS via atom transfer radical polymerization with an activator regenerated through electron transfer. This was realized under dilute monomer concentration conditions (2% monomer content) using an ultra-low copper catalyst concentration, which could be reduced to as low as 1.7 ppm. Guo et al. [72] demonstrated that, at a 20% (mass fraction) monomer loading, monodisperse poly(methacrylic acid–divinylbenzene) microspheres endowed with lauryl, epoxy, hydroxyl, and carboxyl functional groups could be prepared via solvothermal precipitation copolymerization, with a microsphere yield exceeding 94%. While tetrahydrofuran was employed as a co-solvent, the SSA of the microspheres exceeded 400 m^2^/g. Nevertheless, precipitation polymerization demands a low monomer concentration (2~5%), resulting in a sluggish reaction rate and low microsphere yield. Integrating precipitation polymerization with other technologies to further lower production costs, streamline production processes, and scale up production capacity is a future research focus that has garnered significant attention [73].

##### Dispersion Polymerization

**Figure 4:**
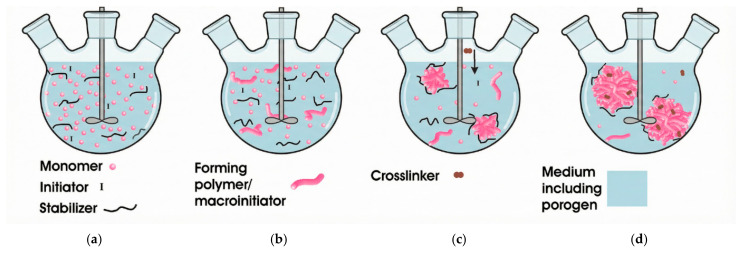
Flowchart of the decentralized aggregation method (the dispersion polymerization process, as depicted in the schematic, involves several successive stages: initially, the monomer, initiator, porogen, and polymeric stabilizer are homogeneously dissolved in the dispersion medium (**a**); upon initiation, soluble oligomers begin to form (**b**); as polymerization progresses to approximately 1% monomer conversion, these growing chains precipitate out of the solution upon reaching a critical length, forming primary nuclei that are immediately stabilized by the polymeric stabilizer; a cross-linker may be introduced at this nucleation stage if required (**c**); subsequently, the stabilized particles continue to grow predominantly by absorbing residual monomer and oligomers from the surrounding medium (**d**)).

Dispersion polymerization is a type of free radical polymerization and a special type of precipitation polymerization [74]. It was proposed and gradually developed by ICI Company in the United Kingdom in the 1970s to overcome the disadvantage of the low concentration of the dispersed phase during the film formation of vinyl coatings and acrylate coatings [75]. The schematic illustration of the stages in dispersion polymerization is presented in Figure 4a–d.

At the beginning of the reaction, the system is homogeneous, and monomers, initiators, porogens, dispersants, etc., are all dissolved in the reaction medium; at a specific stage of the reaction, the produced oligomer molecules tend to precipitate out of the system, yet they can still dissolve in the reaction medium initially; as the chain length of the oligomer molecules escalates, the oligomers start to precipitate from the reaction medium—and this constitutes the difference from conventional precipitation polymerization. The precipitated polymers are not powdery or blocky polymers, but exist in the form of small particles and are suspended in the medium with the help of stabilizers. If it is necessary to prepare microspheres with cross-linked structures, cross-linking agents are generally added at this stage. Then, the nucleated particles continue to capture monomers and oligomers from the reaction medium for growth, and the micro-nuclei continue to grow through polymerization reactions until the reaction terminates.

The nucleation mechanism of dispersion polymerization is relatively complex. At present, the nucleation mechanisms reported in the literature mainly include micellar nucleation, homogeneous nucleation, aggregation nucleation, and coagulation nucleation [76]. Among them, the widely recognized and accepted nucleation mechanisms are the oligomer precipitation mechanism (homogeneous nucleation) proposed by Tseng et al. [77] and the graft copolymer coalescence (micellar nucleation) mechanism proposed by Lok et al. [78]. The graft copolymer coalescence mechanism holds that the dispersant forms a graft copolymer through the reaction of active hydrogen sites with oligomers, which then “anchors” on the surface of polymer particles to prevent flocculation and coalescence during the early nucleation stage. In the early years, some researchers focused on studying the influence of dispersants in dispersion polymerization. A block copolymer consisting of polystyrene and poly(L-glutamic acid) was chosen by Itoch et al. [79] as a mixed dispersant in the dispersion polymerization of styrene, and the effects of the initiator concentration and monomer concentration on the polymerization conditions as well as the microspheres were investigated. Finally, polystyrene microspheres with a diameter of 0.72 μm and a narrow PSD were prepared. This study also opened up new ideas for the selection of dispersants in dispersion polymerization. Lü et al. [80] employed fatty alcohol polyoxyethylene ether (AEO-9) as the co-stabilizer and polyvinylpyrrolidone (PVP) as the steric stabilizer and fabricated quaternary ammonium salt-containing PS particles via two-stage dispersion polymerization in an ethanol/water mixed system (mass ratio 80:20). During the second stage of polymerization, N’-dimethyl-N-n-dodecyl-N-2-methacryloyloxyethyl ammonium bromide (QDMDB) served as the comonomer, enabling the fabrication of functional polystyrene microspheres with a PSD of less than 3%. Liu et al. [81] achieved the successful synthesis of highly monodisperse microspheres derived from poly(benzyl methacrylate) for the first time. This was accomplished via the introduction of the cationic surfactant cetyltrimethylammonium bromide (CTAB), with PVP employed as a stabilizer to inhibit particle agglomeration and CTAB utilized to facilitate dispersion polymerization. By regulating the concentrations of CTAB and monomers, the microsphere size could be tuned within the range of 1.0~10.0 μm, exhibiting excellent monodispersity. Wang et al. [82] synthesized polydodecyl acrylate via reversible addition–fragmentation chain transfer (RAFT) polymerization, employing 2-cyano-2-propyl-dithiobenzoate as the chain transfer agent and dodecyl acrylate as the raw material. Subsequently, with polydodecyl acrylate serving as the stabilizer, they successfully prepared poly(ε-caprolactone) microspheres with a narrow PSD and a particle size spanning from 0.5 to 1.5 μm through ring-opening dispersion polymerization. They also investigated the impacts of the molar mass of polydodecyl acrylate, the reaction temperature, and the volume ratio of the mixed solvent (i.e., 1,4-dioxane/heptane) on the APS and PSD of the microspheres. By carefully controlling the synthesis conditions, microspheres with a PSD of 1.09 (Dw/Dn) can be obtained. The APS of poly(ε-caprolactone) microspheres declines as the MW of polydodecyl acrylate increases, and rises with the elevation of the relative proportion of 1,4-dioxane. Meanwhile, the homogeneity of the microspheres deteriorates as the polymerization temperature increases.

Dispersion polymerization has the advantages of a simple process and a widely applicable monomer range. This constitutes an efficient approach for fabricating uniform microsphere particles that is well suited for both scientific research and industrial development. Fu et al. [83] pointed out in a recent review that, compared with seed swelling polymerization, which relies on multiple steps and intricate process control, dispersion polymerization offers a simpler formulation design and more straightforward scale-up, and is therefore regarded as a more efficient and practical route for constructing highly monodisperse porous microsphere supports. However, there are still many problems to be solved in the use of dispersion polymerization, such as the inability to prepare microspheres with a cross-linking degree greater than 6% [84], the destruction of polymerization balance when copolymerizing with polar monomers, and the difficulty in preparing polymer microspheres with a high content of functional groups [85]. In the future, it is necessary to further understand the dispersion system, solve these current problems, and develop new ideas.

##### Seed Polymerization

Seed polymerization was first proposed in 1948 [86] and is currently the most commonly used method for monodisperse porous polymer particles in liquid chromatography. The seed swelling method requires using monodisperse small particle size microspheres prepared using processing molding methods or other polymerization molding methods as seed microspheres, then swelling them with monomers, cross-linking agents, and inert components and removing the porogen after the reaction to generate porous microspheres [87,88]. Based on the variations in the swelling procedures of seed spheres, the seed swelling method can be categorized into the one-step, two-step, multi-step, and dynamic swelling method.

**Figure 5:**
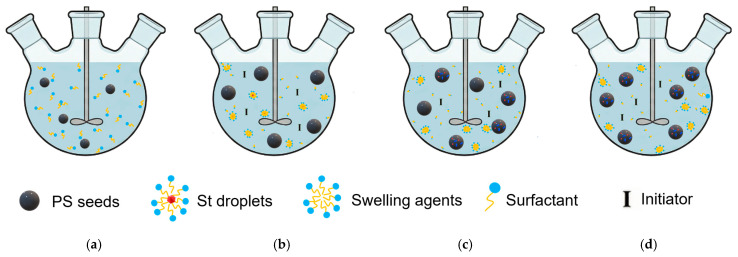
Flowchart of the two-step seed swelling method (the schematic for preparing porous particles through seeded suspension polymerization involves two consecutive stages: first, submicron seed particles are synthesized via emulsion or dispersion polymerization of styrene (**a**); in the second stage—the suspension polymerization proper—the seeds undergo sequential swelling (**b**): initially with an activator such as dibutyl phthalate, and subsequently with a mixture containing fresh monomer, cross-linker, initiator, and porogenic agents (**c**); polymerization of this swollen system results in the formation of monodisperse particles of increased size and porosity (**d**)).

The two-step swelling method was invented by Ugelstad et al. [89,90] in the 1970s. The seed spheres first absorb water-insoluble low-MW compounds to improve the swelling degree, then swell with monomers, initiators, and porogens, and heat to initiate polymerization. The schematic illustration of its synthesis process is presented in Figure 5a–d.

Microspheres fabricated via the two-step swelling method typically exhibit favorable dispersion properties and large particle sizes, making them suitable for subsequent functionalization; yet, their preparation process is rather complex and time-intensive. Tuncel [91] first synthesized a series of monodisperse PS seed spheres with particle sizes spanning from 1.9 to 7.5 μm through dispersion polymerization. Subsequently, monodisperse porous PS-DVB microspheres with particle sizes between 5 and 21 μm were fabricated via the two-step swelling method. He also systematically examined the implications of seed sphere particle size and MW, initiator concentration, and activator and monomer dosages on the particle size and pore structure of the resulting microspheres.

The one-step swelling method was a new swelling technology proposed by Ogino et al. [92] in 1995. Its process flow is mainly divided into two types: the first is that the seed spheres first form a dispersion in water, then stir and swell at a constant speed in a uniform solution of swelling agent, cross-linking agent, initiator, and dispersant, and then heat to initiate polymerization; the second process is that the seed sphere solution is first dispersed in the dispersant solution, then the water/oil phase monomer uniform emulsion containing the dispersant is slowly added in stages and polymerized after swelling. Different from the two-step method, the one-step swelling process is relatively simple, eliminating the use of swelling agents and directly realizing the activation and swelling of seed microspheres through swelling monomers. The swelling and polymerization time are greatly shortened. As the seed spheres are not activated by swelling agents, the particle size of the resulting particles is comparatively small, and the precise regulation of polymerization conditions is necessary to achieve monodisperse particles.

The dynamic swelling method (DSM) was proposed by Professor Okubo [93] in 1991. This method uses monomers, porogens, initiators, and a certain water-soluble solvent as the medium in the seed sphere dispersion system, then steadily adds water to the system to diminish the solubility of monomers in the solvent medium so that the monomers enter the seed spheres for swelling and then heat to polymerize. This method can prepare monodisperse polymer microspheres with a diameter greater than 5 μm without adding swelling agents. Okubo et al. [94] used 1.9 μm polystyrene seed spheres to adsorb DVB through the dynamic swelling method and prepared monodisperse PS-DVB microspheres with an APS of 4.3 μm after reaction. The key technology of the DSM is how to add water to the reaction system slowly and uniformly without local excessive water concentration leading to an uneven swelling ratio, which requires some special technologies, such as adding water through a semi-permeable membrane, combining water in a certain substance to release slowly, adding water using water vapor, etc. Therefore, they later investigated the influence of the water addition rate on the monodispersity of polymer particles [95].

**Figure 6:**
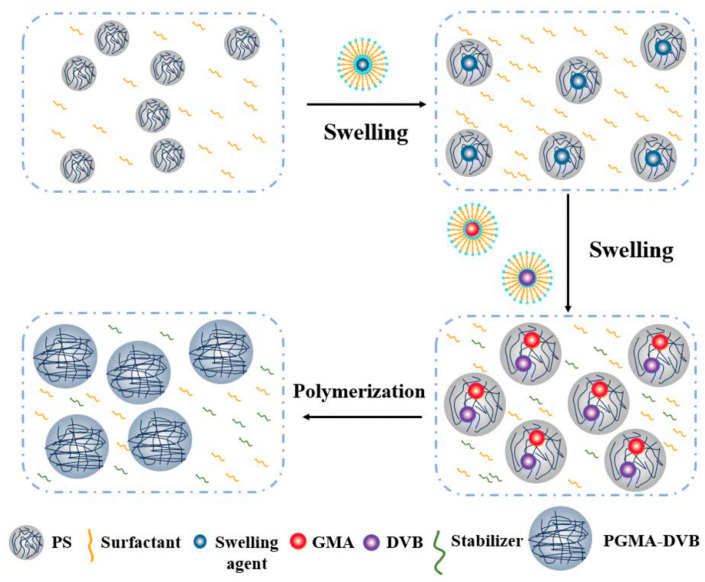
Synthesis of PGMA-DVB microspheres via two-step seed swelling polymerization. Reprinted with permission of Royal Society of Chemistry from [104].

Owing to the restricted swelling capacity of seed spheres in the one-step swelling method, it poses challenges for preparing large-particle-size polymer microspheres. While the multi-step swelling method enables the acquisition of large-particle-size microspheres, new small particles tend to form during repeated swelling and polymerization processes, making the preparation of monodisperse microspheres difficult. Consequently, these two methods are presently less employed. Although DSM can prepare highly monodisperse microspheres, it has a high technical difficulty, high requirements for reaction instruments, and high preparation costs. The two-step swelling method incorporates sphere-activating compounds, enabling the fabrication of monodisperse porous microspheres spanning a broad particle size spectrum, with relatively stable and straightforward operational procedures. This approach has now become the most pivotal method for preparing monodisperse porous microspheres, garnering widespread preference among numerous researchers [96]. For example, in recent years, Tian et al. [97] used polystyrene-4-vinylbenzenesulfonate sodium microspheres as seed microspheres, styrene as a swelling monomer, and divinylbenzene as a cross-linking agent to obtain a series of monodisperse polydivinylbenzene/glycidyl methacrylate (GMA) copolymer microspheres, both solid and hollow. Liu et al. [98] used poly(GMA-DVB) microspheres bearing reactive epoxide groups as the support and prepared an anion-exchange stationary phase by constructing quaternary ammonium layers on the surface. They showed that the exchange capacity could be tuned over a wide range by adjusting the GMA fraction in the microspheres and/or varying the number of quaternized layers, providing a practical route for the targeted design of column capacity and selectivity. Cong et al. [99] fabricated monodisperse porous PS-DVB microspheres featuring an APS of approximately 10 μm via a modified two-step seed swelling approach. They systematically probed into the impact of the cross-linking degree on the SSA of the microspheres and ultimately obtained microspheres with a maximum SSA of 338.21 m^2^/g. Yu et al. [100] achieved the successful fabrication of monodisperse porous PS-DVB microspheres through the two-step seed swelling method and conducted an in-depth investigation into the implications of swelling temperature, swelling agents, and cross-linking agents on the porosity of the resultant microspheres. Microspheres with an SSA between 37~464 m^2^/g were prepared by changing the cross-linking degree, confirming that the cross-linking degree has a significant impact on the porosity of microspheres. Xiao et al. [101] initially fabricated low-MW seed microspheres via dispersion polymerization and observed that the APS of the resultant PS seed spheres increased in conjunction with the elevated dosage of the initiator azobisisobutyronitrile (AIBN). They subsequently produced monodisperse PS-DVB microspheres with a large APS of 10 μm. Samatya et al. [102] obtained 2.5 μm GMA seed spheres through dispersion polymerization, then expanded them with low-MW organic activators, and then mixed the monomer mixture. In this way, monodisperse porous microspheres were fabricated through the polymerization of the monomer mixture in the seed particles and then functionalized with triethylamine to obtain ion chromatography stationary phases. The experimental results showed that the stationary phase has a good analysis effects on five common anions, and the number of theoretical plates for F- reaches 12,904 N/m. Xu et al. [103] first synthesized PS microspheres with a good sphericity, smooth surface, and an APS of 3.26 μm, then used the two-step swelling method with DBP, toluene, cyclohexane, N-heptane, and a mixture of toluene and DBP as porogens to prepare monodisperse cross-linked microspheres of about 7 μm, and concluded that the incorporation of a trace amount of toluene enhanced the monomer adsorption capacity of the swollen seed microspheres, thereby rendering the resultant PS-DVB microspheres nearly devoid of fine particles, with the SSA rising to 68.51 m^2^/g. The PGMA-DVB microspheres synthesized by Shen et al. [104] through the two-step seed swelling method showed a clear porous structure and homogeneous particle size, while maintaining a good monodispersity, with an APS of about 5 μm (Figure 6). Zhang et al. [105] initially fabricated PS seed spheres with an APS of 3 μm, and subsequently prepared 5 μm PS-DVB microspheres using these seed spheres. The PS-DVB microspheres exhibited a pore size distribution of 0.06–0.18 μm, an average pore size of 0.1131 μm, a porosity of 78.26%, and a total SSA of 78.34 m^2^/g, demonstrating a high porosity and uniform pore size. Liu et al. [106] initially produced PGMA and PS seed microspheres via the two-step swelling method, then swelled them into four types of porous polymer microspheres. During the seed sphere stage, a micro-compression tester was employed to compress the seed spheres, and the mechanical characteristics of these particles were assessed via the load–displacement curve. It was observed that PGMA seed spheres are more brittle than PS seed spheres. The APS of the monodisperse PS seed microspheres fabricated by Wang et al. [107] was about 3 μm (CV = 4.8%), indicating that the fabricated PS seed spheres have an outstanding monodispersity. The surface of the seed spheres is smooth and undamaged, indicating that the polymerization process is problem-free and the microspheres have a certain mechanical strength. The PSD of porous microspheres originates from the seed microspheres, thus having a decisive influence on the monodispersity of subsequent porous microspheres. The PS-DVB microspheres fabricated via seed swelling exhibit an APS of 6.6~7.2 μm (CV = 8.0%), demonstrating a relatively uniform particle size and favorable monodispersity. Instrumental test results indicate that the as-prepared porous PS-DVB microspheres have an average pore size of 0.17188 μm, a porosity of 75.222%, and a total SSA of 76.143 m^2^/g.

It should be noted that, to avoid the formation of new seeds, it is indispensable to strictly maintain the appropriate synthesis conditions (number of seed particles, stabilizer and initiator concentration) in the seed swelling method so that the PSD of the resultant particles is consistent with the initial seed spheres. Moreover, when preparing porous microspheres using the seed swelling method, each swelling step will amplify the defects of the seed spheres, so the preparation of seed spheres with uniform particle size is crucial.

In summary, for various reasons, except for the seed swelling method, other methods are relatively rarely used in the fabrication of chromatographic stationary phase matrices, and the seed swelling method is the most widely used. 

Table 4 lists the microspheres obtained through different preparation methods.

## 3. Functionalization of Stationary Phase Matrices

**Figure 7:**
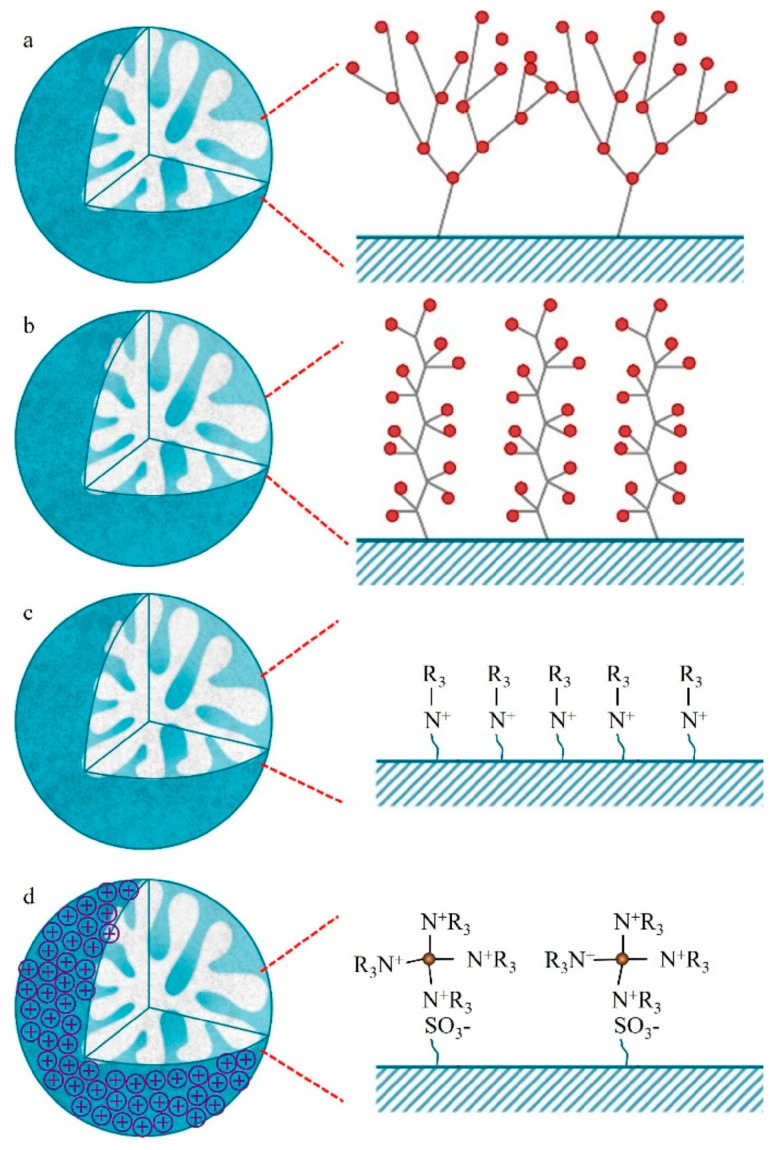
Common functionalization modification methods for ion chromatography stationary phases: (**a**) hyperbranching method; (**b**) surface grafting; (**c**) chemical derivatization; (**d**) latex agglomeration.

To achieve the efficient separation of target ions, polymer microspheres need to be functionalized to introduce specific ion exchange groups on their surface or in their pores. There are two main approaches to preparing functionalized microspheres: one is to directly introduce functional monomers for copolymerization during the polymer synthesis stage; the other is to post-modify the formed polymer matrix. Taking PS-DVB microspheres as an example, the high reactivity of their benzene rings facilitates the introduction of various functional groups through substitution reactions such as chloromethylation and sulfonation [109,110,111]; in addition, functional monomers can also be added during polymerization to optimize the hydrophilicity and selectivity of the matrix [26]. At present, most of the reported ion chromatography stationary phase packings adopt the latter, that is, using the prepared polymer microspheres as matrix carriers and further modifying the matrix through chemical derivatization [112,113,114], surface grafting [108,115,116], latex agglomeration [117,118,119], hyperbranching modification [98,120,121], and combinations of the above modification methods [122,123,124] on the polymer surface. Although some researchers have classified the surface coating method as a non-covalent modification method for anion exchange chromatography stationary phases [14], it is mainly used to directly coat ionic surfactants on the surface of inorganic matrices, such as reversed-phase liquid chromatography stationary phases, to construct functional layers, and the coating is fixed by hydrophobic interactions, resulting in the strong hydrophobicity of the stationary phase [125]. Moreover, there have been few studies in recent years, so it is not elaborated on as a separate chapter in this paper. These other methods lay the foundation for the high-selectivity and high-sensitivity analysis of anions in complex environmental samples by precisely regulating the type, density, and distribution of functional groups. The schematic diagram of several common functionalization modification methods for polymer-based ion chromatography stationary phases is shown in Figure 7.

### 3.1. Chemical Derivatization

Chemical derivatization undergoes a direct reaction with functional reagents via reaction sites on the matrix surface (e.g., the benzene ring of PS-DVB), enabling the modification of numerous functional groups on the stationary phase and thus yielding chromatographic packings with a high exchange capacity. This approach typically encompasses classic organic reactions including chloromethylation, amination, sulfonation, Friedel–Crafts alkylation/acylation, and carboxylation.

#### 3.1.1. Chloromethylation

Chloromethylation is the process of introducing chloromethyl groups onto the benzene ring by substituting hydrogen atoms on the benzene ring with chloromethyl ether under the action of catalysts such as zinc chloride. Chlorine has a high activity and can react with many reagents to further introduce other functional groups. The chloromethylation reaction is one of the most widely used and studied reactions in the chemical modification of mainstream polymer matrix PS-DVB microspheres. Due to the strong toxicity and carcinogenicity of chloromethyl ether, many researchers are committed to improving the chloromethylation method or developing new methods. One is to use long-chain chloromethyl ethers (such as chloromethyloctyl ether, 1-chloro-4-chloromethoxybutane, 1,4-bis(chloromethoxy)butane, etc.) instead of chloromethyl ether. Long-chain chloromethyl ethers have high boiling points and a low volatility, and no carcinogenicity has been found so far. The second is to copolymerize chloromethylstyrene with styrene and divinylbenzene to directly introduce chloromethyl groups into microspheres [126]. The limitation of this strategy is that chloromethylstyrene is comparatively expensive, which increases the production cost of microspheres, and the addition of p-chloromethylstyrene affects the monodispersity of microspheres, so its application is also limited. Table 5 lists some classic chloromethylation methods of PS-DVB microspheres.

#### 3.1.2. Friedel–Crafts Alkylation/Acylation

The Friedel–Crafts alkylation/acylation of porous PS-DVB microspheres is the process of introducing alkyl and acyl groups onto the benzene ring by substituting hydrogen atoms on the benzene ring with alkyl halides, acyl halides, acid anhydrides, etc., under the action of catalysts such as anhydrous aluminum trichloride. The introduction of chloroacetyl groups into PS-DVB microspheres after chloroacetylation can replace the chloromethylation reaction. This reaction not only avoids the use of carcinogens (e.g., chloromethyl ether) but also enables the acyl group to exert a passivating effect on the benzene ring, preventing the occurrence of multi-substitution reactions. Furthermore, compared to using chloroformyl chloride for chloromethylation, it can ensure that the length of the connection chain between the group and the substrate surface is no longer merely 1. This might cause a change in the selectivity of the fixation with respect to the analyte. Table 6 and Table 7 list some classic alkylation/acylation methods of PS-DVB microspheres, respectively.

#### 3.1.3. Nitration

PS-DVB microspheres can react with a mixture of concentrated nitric acid and concentrated sulfuric acid at a certain temperature to introduce nitro groups onto the benzene ring. The nitro groups can be reduced to amino groups, which can be used as intermediates to convert into other functional groups. Corradini et al. [142] nitrated highly cross-linked PS-DVB microspheres, then reduced the nitro groups to amino groups with stannous chloride and hydrochloric acid, and further reacted with iodomethane to introduce quaternary ammonium groups onto the microsphere surface. The modified microspheres can be used for the separation of sugars. Table 8 lists some classic nitration methods of PS-DVB microspheres.

However, the limitations of chemical derivatization technology are not only at the operational level, but also in its inherent “extensive” characteristics, which are inconsistent with the requirements of modern chromatography for precise structure control. On the one hand, its reaction sites (such as benzene rings) are randomly distributed in the matrix, and the density and position of functional group modifications are difficult to accurately control, resulting in uneven surface chemical properties of the stationary phase and great challenges in batch reproducibility. On the other hand, violent chemical reactions irreversibly change the bulk structure of the polymer matrix (such as cross-linking degree, pore size distribution), which may increase the mechanical strength, but often at the expense of mass transfer efficiency and column efficiency [144]. Specifically, the disadvantages of chemical derivatization are particularly prominent in the fabrication of anion exchange stationary phases. Although chloromethylation is efficient, it relies on highly toxic chloromethyl ether, exposing the lack of safety and green chemistry in traditional methods, which is no longer in line with modern laboratory specifications. The nitration-reduction route has complex side reactions, and it is difficult to completely reduce nitro groups, resulting in a complex surface chemical environment of the obtained stationary phase. The remaining nitro groups or intermediates become uncontrollable secondary adsorption sites, directly damaging selectivity and column efficiency. In contrast, the acylation reaction using acetic anhydride has relatively mild reaction conditions and an easy control of by-products. Its value not only lies in avoiding the above problems, but also in that the generated ketone groups can be used as a general and highly active platform for subsequent “click chemistry” or hyperbranching modification [145], realizing the transformation from “terminal modification” to “iterative functionalization” [7].

Therefore, the decline of the chemical derivatization method is not only due to its cumbersome operation or use of toxic reagents, but also fundamentally because it cannot meet the core requirements of high-performance chromatography for the surface chemical precision, structural uniformity, and functional designability of stationary phases. In the past two decades, its application has been relatively limited, and it has been gradually replaced by more precise and controllable mainstream strategies, such as surface grafting, latex agglomeration, and hyperbranching. However, there are still a small number of commercial columns with high selectivity, which need to be further studied by researchers. The anion exchange stationary phases prepared through the chemical derivatization method summarized in Table 5, Table 6, Table 7 and Table 8, from the current perspective, are more like a “historical map” showing the relationship between the type of functional groups and basic performance of stationary phases, and their academic value lies in revealing the context of early exploration rather than representing the cutting-edge direction of current technology.

### 3.2. Surface Grafting

**Figure 8:**
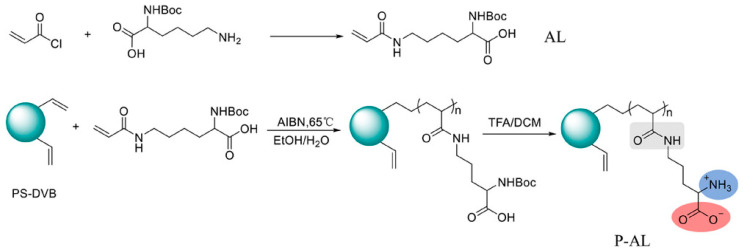
Synthesis route of the zwitterionic stationary phase. Reprinted with permission of John Wiley & Sons from [151].

Surface grafting refers to connecting pre-synthesized or in situ polymerized functional polymer chains to the surface of matrix microspheres through covalent bonds, thereby preparing new composite materials with multiple properties. This method can keep ion exchange functional groups at a distance from the hydrophobic matrix core, effectively weaken non-specific adsorption, and enhance separation efficiency. The matrix material can be silica or organic polymer microspheres, but its surface needs to have or can be introduced with groups that can participate in polymerization reactions. Based on the growth pattern of polymer chains, surface grafting falls into three strategies, namely “grafting-to”, “grafting-through” and “grafting-from” [146]. Among them, “grafting-from” has become the mainstream method due to its ability to achieve a high grafting density [14]. Atom transfer radical polymerization (ATRP), owing to its excellent controllability over the chain growth process and capability to immobilize dense functional groups, has been comprehensively adopted among the diverse array of grafting techniques [147]. Nevertheless, ATRP necessitates a series of reaction steps to introduce initiator groups, a process that could compromise the purity of the final product [124]. Moreover, it is hampered by steric hindrance effects, making it challenging to achieve a high grafting density. Apart from ATRP, free radical polymerization also serves as a viable grafting approach. Although its polymerization process has poor controllability, it has the advantage of not requiring complex modifications of the matrix, and can directly react in a system containing resin, monomers, and initiators to bond polymer layers on the surface of microspheres. It should be noted that, if cross-linking monomers are used, the reaction system is prone to gel formation, resulting in microspheres being dispersed in it, which is not suitable for surface grafting. Studies have shown that divinylbenzene (DVB) often retains some reactive side double bonds during copolymerization, which can be used as sites for subsequent functionalization. Using this characteristic, researchers have designed a variety of new ion exchange stationary phases. For instance, Yang et al. [148] immobilized allyl glycidyl ether (AGE) on the surface of PS-DVB microspheres via free-radical copolymerization, and then introduced quaternary ammonium groups by conducting a ring-opening reaction with N-methyldiethanolamine (MDEA), thus successfully fabricating a hydroxide-resistant anion stationary phase, denoted as P@A-M. This stationary phase exhibited an excellent hydroxide selectivity, satisfactory separation performance for conventional inorganic anions (with a resolution higher than 1.5), and an outstanding operational stability (the relative standard deviation of retention time was less than 1.13%). Kaltz et al. [149] anchored VB-DEMA onto PS-DVB through free-radical polymerization, and the resultant anion exchange stationary phase was capable of separating seven common inorganic anions within 13 min. In another study, Yang et al. [150] anchored the quaternary ammonium monomer DMC onto the surface of GMA-DVB porous microspheres via the pendant double bonds of DVB, combined with the hydrolysis of epoxide groups to enhance hydrophilicity, thereby constructing a new type of anion-exchange material with a tunable capacity, high separation efficiency, and rapid separation capability for common inorganic anions. Shen et al. [151] fabricated a novel polymer-based polar stationary phase by immobilizing modified lysine onto PS-DVB microspheres with a cross-linking degree of 55% and an ASD of 5.5 μm via free-radical polymerization. They also verified that the incorporation of two-dimensional special ionic groups and amide groups could remarkably enhance the surface hydrophilic properties of the PS-DVB microspheres. The obtained stationary phase can separate both cationic and anionic analytes, and the column performance did not significantly decrease after stable operation for 8 months (Figure 8).

Dendrimers are a new type of functional polymer materials developed in the 1930s, with perfect dendritic structures [152,153]. Such materials can precisely control their size, shape, structure, and functional group design at the molecular level. Their highly branched structure, uniform monodispersity, spacious internal cavity, and abundant terminal active groups endow dendrimers with unique properties and functions, such as good dispersibility and biocompatibility, outstanding electrical and optical properties, low toxicity, and simple modification [154]. Dendrimers (e.g., PAMAM and PEI) possess three-dimensionally ordered architectures, abundant surface functional groups, and excellent hydrophilicity, rendering them ideal grafting materials. Immobilizing these dendrimers onto the surface of polymer microspheres can efficiently address the issue of insufficient functional group loading arising from the limited SSA of conventional matrix materials. The column capacity and separation efficiency can be precisely adjusted by regulating the molecular chain structure [155,156]. Polyamidoamine (PAMAM) dendrimers are a type of dendrimer materials with ethylenediamine as the core and amide bonds as repeating structural units. Integral-generation PAMAM dendrimers possess abundant terminal amino functional groups that tend to undergo protonation readily. Based on this characteristic, the research group [156,157,158,159,160,161,162] has found that PAMAM dendrimers, with ethylenediamine as the core and amide bonds as repeating structural units, are highly branched [163] and have been the most widely used among the various dendrimer materials so far [164]. The current literature studies [157,165,166,167] have shown that PAMAM adsorbent materials based on ionic interactions exhibit an excellent performance in the adsorption of inorganic anions, acid radical ions, organic dyes, organic pesticides, and biologically active substances, showing broad application prospects. Researchers utilized PAMAM dendrimer materials for fabricating novel ion chromatography stationary phase fillers via chemical grafting, as well as latex agglomeration techniques, thereby attaining certain phased accomplishments [156,161,162]. Guo et al. [161] immobilized PAMAM onto the PS-DVB-GMA matrix and functionalized the terminal amino groups with 1,4-butanediol diglycidyl ether (1,4-BDDGE), thereby fabricating dendritic stationary phases possessing an elevated density of quaternary ammonium groups. By altering the generation of PAMAM, the retention performance of the stationary phase is tunable, enabling the realization of high-purity and structurally intact dendritic grafting, thus circumventing the structural defects that might be induced by surface modification. In addition, the stationary phase also has excellent biocompatibility. However, PAMAM also has drawbacks such as unfavorable cost, inadequate alkali resistance, and low column efficiency. To further enhance performance, PAMAM was grafted onto the graphite oxide-coated PS-GMA composite particles. The resulting separation for organic weak acid anions and inorganic anions was indeed improved [162].

**Figure 9:**
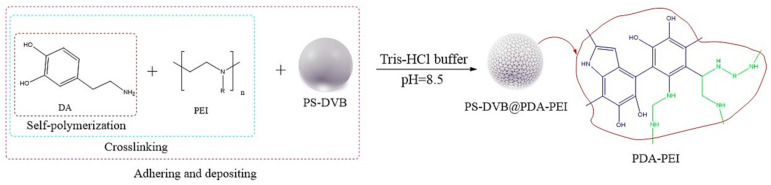
Synthesis of PS-DVB@PDA-PEI microspheres. Reprinted with permission of Elsevier from [173].

PEI, as a hyperbranched polymer possessing a multitude of amino groups in its chain, has a strong hydrophilicity, can lessen non-ionic interactions, and is suitable for constructing stationary phases with high branching degrees [168]. Covalently grafting it onto the surface of microspheres and quaternizing with epoxy compounds can prepare anion exchange stationary phases, and some studies have been carried out [169,170,171]. For example, Yang et al. [172] immobilized PEI onto the GMA-DVB matrix and subsequently carried out a hydrolysis treatment to transform the residual epoxy groups on the surface of the GMA-DVB matrix into hydroxyl groups. Eventually, they conducted the quaternization of PEI using epoxy-containing molecules. Via this approach, the resultant stationary phase accomplished the complete elution of seven commonly encountered inorganic anions in 16 min. Liu et al. [173] deposited a strongly adhesive PDA film on the surface of PS-DVB microspheres and subsequently covalently anchored PEI onto the PDA coating. After that, they utilized the abundant primary and secondary amino groups on the PEI chains to perform alternating reactions with MA (4% aqueous solution) and BDDGE (10% aqueous solution), thus introducing quaternary ammonium groups and further endowing the material with anion exchange capability. The resultant stationary phase exhibited a favorable separation performance for iodate, chromate, and six organic weak acids (Figure 9).

**Figure 10:**
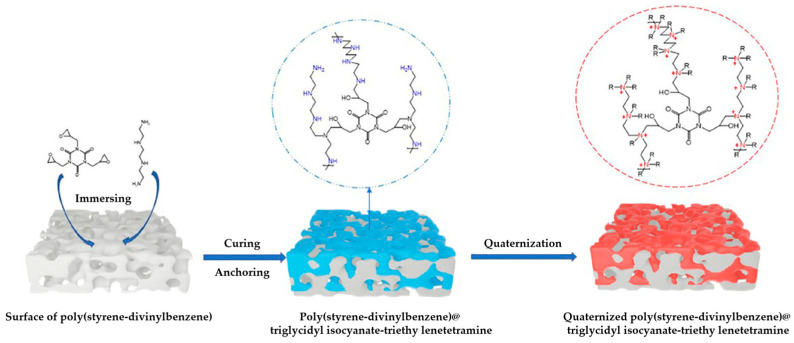
The schematic diagram of the synthesis route of quaternized PS-DVB@triglycidyl isocyanate–triethylenetetramine. Reprinted in modified form with permission of John Wiley & Sons from [174].

Recently, there have been studies on grafting COF onto polymer matrices instead of previous linear polymer chains or dendrimers, which has achieved a leap from “grafted chains” to “grafted frameworks”. The structural order and functional designability of the functional layer have been greatly enhanced. Liu et al. [174] deposited a triglycidyl isocyanate network coating onto the surface of PS-DVB via an amine-curing reaction, thereby fabricating melamine-DVB@triglycidyl isocyanate–triethylenetetramine composite microspheres. The amino and epoxy groups in triglycidyl isocyanate–triethylenetetramine endow PS-DVB with a superior reactivity, allowing the material to undergo quaternization under mild reaction conditions and thus be converted into anion exchange chromatography stationary phases (Figure 10). Later in another study [175], they prepared PS-DVB@p-phenylenediamine-1,3,5-triformylphloroglucinol composite microspheres through a one-step in situ growth approach. Before the quaternization process, the as-prepared PS-DVB@p-phenylenediamine-1,3,5-triformylphloroglucinol composite microspheres were soaked in 30 mL of ethanol solution with 0.4 g of sodium borohydride incorporated, and were incubated at room temperature for 24 h. Subsequently, the reduced composite microspheres were subjected to alternating reactions with 3% MA aqueous solution and 6% 1,4-butanediol diglycidyl ether solution, with the quaternization process being accomplished by regulating the duration of repeated reactions with the two aforementioned reagents.

Compared with the chemical derivatization process, surface grafting provides the possibility of obtaining thin film structures because the grafted layer is affixed to the surface without permeating the pores of the substrate particles. This thin-layer functional structure can effectively improve the column efficiency. Table 9 lists the differences between chemical derivatization and surface grafting modification. However, compared with emerging methods such as hyperbranching, it still has certain limitations in the number of functional group modification layers.

### 3.3. Latex Agglomeration

**Figure 11:**
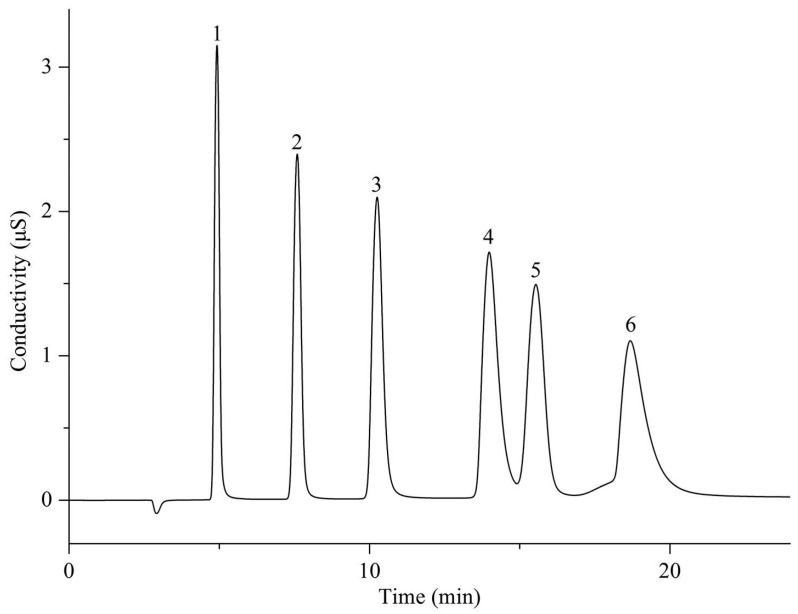
Separation of six conventional anions using a septuple quaternized g-C_3_N_4_ nanosheet-agglomerated PS-DVB anion-exchange chromatographic column. Eluent: 10 mmol L^−1^ NaOH; flow rate: 1.0 mL min^−1^; injection volume: 25 μL; detection: suppressed conductivity detection; peaks: (1) fluoride, (2) chloride, (3) nitrite, (4) bromide, (5) nitrate, (6) sulfate. Reprinted with permission of Elsevier from [177].

Since latex agglomeration was proposed by Small et al. in 1975, it has become one of the most comprehensively used stationary phase construction strategies in commercial anion exchange columns. This method attaches latex particles with a particle size of tens to hundreds of nanometers to the surface of matrix microspheres through electrostatic and van der Waals forces to form a shell structure of agglomerated nanoparticles. Its separation mechanism is primarily dependent on the Donnan exclusion effect, which can efficiently preclude inorganic ions from penetrating the inert hydrophobic domain of the stationary phase, thereby markedly mitigating non-specific adsorption [14]. The core advantage of this method resides in its superior adjustability: the exchange capacity of the chromatographic column is capable of being accurately modulated by altering the dimensions of latex particles and matrix microspheres; the selection of latex particles with an ASD below 0.1 μm enables the attainment of maximal separation efficiency; and the homogeneous coating of monodisperse latex particles can eliminate the interference of exposed negative charges on anion separation. Latex agglomerated stationary phases usually have a thin functional layer, which makes the ion exchange process fast and efficient. Its selectivity mainly depends on the cross-linking degree of latex, the properties of the base polymer, and the structure of quaternary ammonium groups. For instance, Sun et al. [176] fabricated epoxy-containing AGE-ST copolymer latex via the soap-free emulsion polymerization of AGE and styrene. After undergoing quaternization, the latex was immobilized on the surface of sulfonated PS-DVB microspheres, thus successfully producing a stationary phase that exhibits outstanding separation achievement for conventional anions and organic acids. Liu et al. [177] used positively charged graphitic carbon nitride nanosheets as functional units and physically agglomerated them onto the surface of negatively charged (e.g., pre-sulfonated) PS-DVB microspheres through electrostatic attraction and other forces to form a nanosheet shell. It was demonstrated that PS-DVB hybridized with g-C_3_N_4_ nanosheets exhibited a favorable reactivity owing to the existence of terminal amino groups. After column packing, the resultant stationary phase was able to realize the baseline separation of six common anions and four monovalent organic acids (Figure 11). Wang et al. [119] developed a new polymer-based stationary phase using macroporous PS-DVB resin: the PS-DVB beads were first sulfonated to introduce negatively charged sites, and a cationic polyelectrolyte (PEPI-DMA) was then assembled/immobilized onto the particle surface via electrostatic interactions, yielding an anion-exchange material suitable for ion chromatography. This coating strategy modified the pore structure parameters without compromising the morphology of the support (increased specific surface area and pore volume, while reducing the average pore width), and endowed the column with the effective separation of multiple inorganic anions, with I^−^ being clearly resolved from coexisting ions.

**Figure 12:**
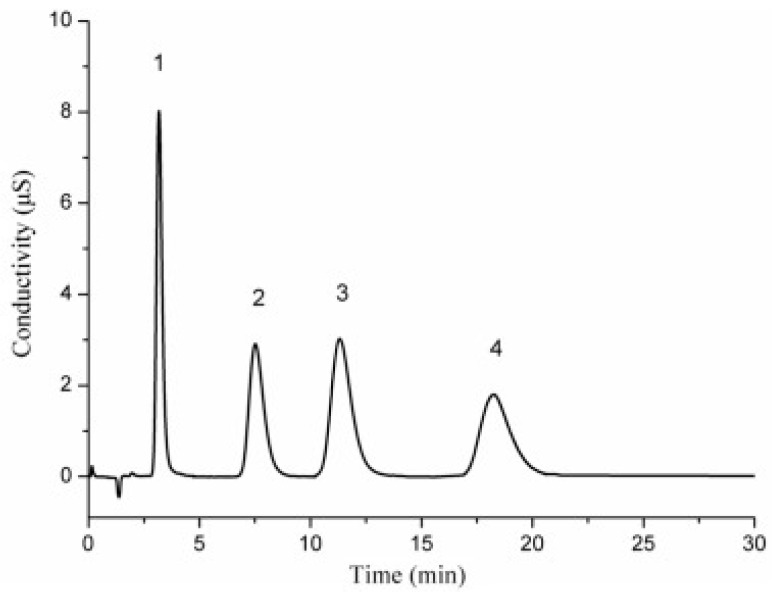
Separations of four polarizable anions on Q_1_HCS column. Eluent: 9.0 mmol L^−1^ KOH; flow rate: 1.0 mL min^−1^; injection volume: 25 μL; peaks: (1) thiosulfate, (2) iodide, (3) thiocyanide, (4) perchlorate. Reprinted with permission of Elsevier from [182].

In addition to latex agglomeration, polyelectrolyte agglomerated anion exchange stationary phases are agglomerated pellicular stationary phases prepared by adsorbing the functional groups of cationic polyelectrolyte backbones onto the surface of negatively charged matrices through electrostatic interaction [178,179]. Cationic polyelectrolytes are water-soluble polymer compounds whose segments contain a large number of ionizable groups, thus having the dual structural characteristics of small-molecule charged groups and polymer long chains [180]. In particular, quaternary ammonium salt polymer compounds have become a research hotspot in recent years, developing rapidly with many types, and are an important class of functional polymer polyelectrolytes. Previous relevant reviews by researchers have independently classified it as a modification method [14], but, considering that it is similar to the stationary phase form prepared through latex agglomeration, it is classified as one category in this paper. However, due to the high charge compensation of amino polymer chains and the easy conformational changes in the functional layer during use, their stability and efficiency are usually lower than those of latex agglomerated stationary phases. To improve performance, Wang et al. [181] synthesized viologen polyelectrolyte cationic functional groups and agglomerated them onto sulfonated PS-DVB to prepare anion exchange stationary phases with a good stability and acid–base resistance. It can achieve the rapid separation and analysis of six common anions within 8 min, with a linear range of 0.5–50 mg/L and a correlation coefficient (r) of 0.9992. After long-term use, the retention time of sulfate has no significant change. Another study [182] modified hydrothermal carbon nanospheres (HCSs) with quaternized polyelectrolytes and then agglomerated them onto PS-DVB, but the quaternization process reduces the thermal stability of HCSs to a certain extent. This stationary phase is capable of efficiently resolving commonly seen inorganic anions, monocarboxylic acids, carbohydrates and polarizable anions within 20 min (Figure 12). Moreover, the superior hydrophilicity of the HCS surface endows all analytes with excellent peak symmetry.

In general, as a mature and controllable stationary phase modification technology, latex agglomeration provides a stable and reliable tool for the efficient and high-selective analysis of anions in water environments through the flexible combination of matrices and latex.

### 3.4. Hyperbranching

Hyperbranching is an effective strategy for preparing chromatographic packings with a high exchange capacity by constructing three-dimensional branched structures on the surface of porous matrices to introduce high-density functional groups [121]. The methodology of stepwise polymer growth on polymer matrices represents one of the most comprehensively adopted modern stationary phase architectures for commercial chromatography columns, including Thermo Fisher AS30 series (Waltham, MA, USA). This method usually first introduces a negatively charged functional base layer on the matrix microspheres (including the outer surface and the inner surface of pores), then adsorbs epoxy–amine copolymers through electrostatic interaction and uses primary amine or multi-epoxy monomers to construct branched reaction sites on the surface. The reaction conditions of this process are mild, which does not damage the matrix structure and can effectively break through the limitation of the number of functional groups of the SSA of microspheres.

Hyperbranched stationary phases are rich in hydroxyl groups on the surface, with a good hydrophilicity, which helps to weaken non-specific adsorption caused by hydrophobic interactions and improve chromatographic peak shape. They are resistant to strong alkaline eluents, have a high chemical stability, and can precisely adjust the ion exchange capacity by regulating the number of branching reactions, thereby customizing stationary phases with different retention characteristics. However, such stationary phases usually have a weak retention capacity for multivalent anions; moreover, owing to the low charge density of quaternary ammonium groups, the ion exchange process may be slow and column efficiency may decrease. Particularly in the analysis of high-concentration hydroxide eluents and large-sized multivalent anions, the cross-linking of hyperbranched structures will limit the alkylation degree and further reduce the charge density. Recent research has revealed that linear functionalized anion exchange stationary phases featuring low cross-linking degrees possess a higher charge density and demonstrate a superior chromatographic performance. Uzhel et al. [120] pioneered a novel approach by first incorporating secondary amine groups onto PS-DVB matrices, followed by constructing hyperbranched polymer layers through alternating reactions with 1,4-BDDGE and MA. The study found that, when the number of modification cycles increased from 3 to 4, the selectivity of the stationary phase was significantly improved; continuing to increase the number of cycles only slightly changed the selectivity, but the exchange capacity was further improved. The stationary phase obtained after three modification cycles had column efficiencies of up to 18,000 N/m and 16,000 N/m for NO_2_^−^ and Br^−^, individually. Zhang et al. [183] used thiol-ene click chemistry to immobilize cysteamine/cysteine on polymer microspheres to prepare aminated matrices and then grafted hyperbranched polycondensates. This method uses 1,4-BDDGE and MA as components for cyclic reactions, and replaces MA with N-methyldiethanolamine in the final cycle to form quaternary ammonium terminals, realizing the goal of preparing stationary phases with an expected capacity through fewer reaction cycles. The anion exchange stationary phase with a three-layer structure can simultaneously separate nine anions within 17 min only by isocratic elution, with a column efficiency of up to 16,100 N/m.

**Figure 13:**
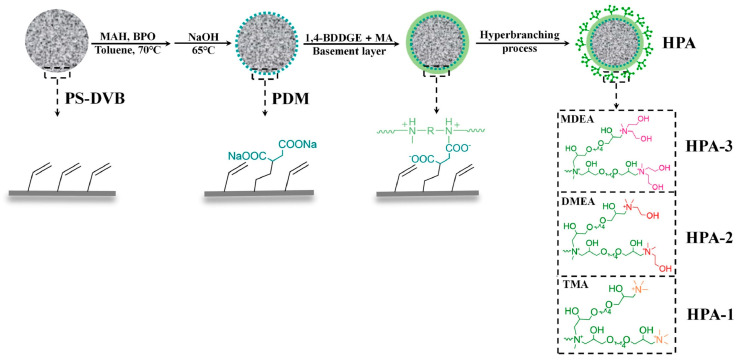
Scheme for the synthesis of a hyperbranched anion exchanger. Reprinted with permission of Elsevier from [186].

Integrating the hyperbranched modification strategy with carbon nanomaterials (e.g., graphene, carbon nanospheres and nanodiamonds) can further enhance the mechanical robustness, selectivity and thermal stability of stationary phases. Zhang et al. [184] coated graphene oxide on PS-DVB microspheres, obtained graphene-coated polymers (G@PS-DVB) after reduction, and then grafted quaternary ammonium groups through PEI and hyperbranching reactions. This stationary phase exhibited an efficient and symmetric separation performance towards diverse anions, and its ion exchange capacity was tunable via regulating the dosage of graphene and the number of hyperbranched layers. These stationary phases have good column efficiency and symmetry for various anionic analytes. Continuous injection experiments show that the combination of the carbon-doped graphene (cdG) layer and anion exchange function has a high chemical stability and good reproducibility. Zhao et al. [123] adopted PEI for the amidation of porous carbon nanospheres, and then conducted hyperbranched modification via the epoxy–amine addition reaction of ethylene glycol diglycidyl ether and MA. Ultimately, they anchored the functionalized carbon spheres onto the PS-DVB substrate via electrostatic forces. As the number of grafting layers increased from 2 to 5, both the hydrophilicity and ion exchange capacity of the stationary phase were enhanced. Additionally, the rigid structure of carbon spheres facilitated mass transfer processes, thus shortening the overall separation time. The column efficiency for seven types of inorganic anions extended from 13,200 to 38,400 N/m. Yao et al. [185] immobilized quaternized nanodiamonds on the surface of sulfonated PS-DVB via electrostatic interaction and then conducted further hyperbranched modification to fabricate a series of composite stationary phases. Among these materials, the stationary phases (LAH-AE2 and AH-AE2) prepared after two modification cycles were able to withstand extreme pH conditions ranging from 1 to 13 and also exhibited a favorable stability and excellent pressure resistance when applied in organic solvents. As the exchange capacity increased, the retention capability of anions was strengthened. Eventually, under the elution conditions of 8 mmol/L sodium carbonate and 8 mmol/L sodium bicarbonate, the baseline separation of seven anions was achieved within 60 min. Shen et al. [186] used PS-DVB microspheres as carriers to graft maleic anhydride to prepare a surface-carboxylated hyperbranched anion exchanger, which was then hydrolyzed to generate carboxylate groups. The obtained stationary phase had a high hydrophilicity, and the theoretical plate numbers of chlorine reached 51,000 N/m (Figure 13).

The hyperbranched modification approach enables the fabrication of stationary phases with a high ion-exchange capacity and excellent selectivity and paves a novel path for the development of mixed-mode separation materials. However, this method still faces problems such as a low grafting rate, low yield, and easy generation of structural defects with the increase in layers [187]. Future research can concentrate on developing new modification methods of carbon nanomaterials to improve grafting efficiency and material structure integrity while retaining the advantages of hyperbranching, so as to promote its wide application in the examination of trace anions in water environments.

In summary, the separation performances of anion chromatography stationary phases with a good performance prepared through these functionalization methods in recent years are listed in Table 10.

## 4. Conclusions and Outlook

The physical and chemical properties of the stationary phase directly determine the key performance parameters of the chromatographic column, which ultimately influence the method’s separation efficiency and analytical speed for target analytes. This review shows that organic polymer matrices have become the mainstream materials for current ion chromatography stationary phases due to their excellent modifiability and mechanical stability. However, it is worth noting that their preparation and functionalization processes still rely largely on empirical exploration. Despite decades of development, in which the performance of polymer stationary phases has been significantly improved, there is no clear consensus in the academic community on whether to prioritize optimizing hyperbranching methods, improving latex agglomeration technology, exploring new chemical derivatization strategies, or committing to synthesizing matrices with a better pore structure, higher SSA, or ordered internal characteristics in the future.

With the progress of synthetic chemistry, the research paradigm in this field is gradually shifting from the early “synthesize first, then explore performance” to “targeted design and synthesis” oriented by application needs. This transformation provides the possibility for targeted material design and performance optimization. Therefore, it is imperative to attain a more in-depth understanding of the separation and adsorption mechanisms of polymer-derived stationary phases at the molecular level in subsequent research. This helps for conducting the virtual screening and design of chromatographic steps with the help of computers, and can introduce data-driven methods such as machine learning and molecular dynamics simulations [190] to optimize the design and performance of stationary phases. In recent years, such rational design has emerged: for example, Zhang et al. [191] predicted the adsorption performance of basic anion resins with styrene skeletons for alginate oligosaccharides through molecular simulation and verified their high adsorption capacity and desorption rate through experiments; Aral et al. [192] used quantitative structure–activity relationship models to clarify the synergistic retention mechanism of hydrophobic, hydrogen bonding, and electrostatic interactions in tris(hydroxymethyl)aminomethane-functionalized mixed-mode stationary phases; Hou et al. [193] conducted a systematic investigation into the adsorptive behaviors of cation exchange resins bearing diverse functional groups toward chitooligosaccharides, laying a theoretical foundation for screening highly selective separation matrices by integrating thermodynamic and kinetic analyses. These works have jointly promoted the paradigm evolution of ion chromatography stationary phase research from empirical trial and error to rational design. Future research on polymer-based anion chromatography stationary phases is expected to advance in the following key directions, thereby expanding the frontiers of selectivity, column efficiency, and application scope.

(1) Rational functional design based on theoretical modeling

Future research should go beyond empirical synthesis and move towards a rational design paradigm. For specific analytes such as glyphosate and short-chain perfluorinated compounds, it is necessary to combine theoretical frameworks such as solvation models, double-layer models, and linear solvation energy relationships with computational tools such as quantum chemical calculations and quantitative structure–activity relationships. This synergy can realize computer-aided screening and optimization before synthesis, including the design of functional monomers, grafting methods, and linkers. Through quantifying the contributions of intermolecular interactions, including electrostatic forces, hydrophobic interactions, and hydrogen bonds, this approach enables the elucidation of the interaction mechanism between analytes and stationary phases at the molecular level. This, in turn, lays a solid foundation for tailoring stationary phases with superior selectivity and column efficiency to meet specific application requirements.

(2) Precision engineering of matrix substrates

The development of modern chromatographic instruments, especially ultra-high performance liquid chromatography systems, places higher requirements on the mechanical stability and kinetic performance of stationary phases. Future work should focus on the precision engineering of PS-DVB matrices themselves: (1) synthesize polymer microspheres with uniform particle size and controllable pore structure (such as pore size distribution and connectivity) to enhance column efficiency; (2) utilize molecular dynamics simulations to visualize the diffusion path of analytes, thereby guiding the design of optimal pore structure to minimize mass transfer resistance; (3) maximize the accessible SSA for target analytes while ensuring structural robustness. This series of strategies aims to prepare matrix materials perfectly compatible with modern chromatographic systems.

(3) Integration of advanced porous and functional materials

Integrating new porous materials into stationary phases contains transformative opportunities. Materials including porous organic cages, COFs, and MOFs are capable of providing highly ordered and tunable pore structures, as well as abundant surface chemical properties. Introducing them as composite materials or coatings can bring unique selective recognition and interaction sites. In addition, ionic liquids with flexibly designable cations and anions can also provide a multi-interaction platform, including hydrophobicity, hydrogen bonding, and ion exchange through grafting. Exploring the combination of these materials with polymer matrices is expected to produce stationary phases with unprecedented separation performance.

(4) Development and application of mixed-mode stationary phases

Mixed-mode stationary phases can synergistically utilize assorted interaction mechanisms such as hydrophilic interactions, ion exchange, and reversed-phase interactions in a single material, and have shown an excellent stability, reproducibility, and separation efficiency in complex sample analysis. Their successful applications in the analysis of traditional Chinese medicine components, environmental pollutants, and food pollutants have confirmed their great practical value. Future synthetic research should strategically utilize this principle, no longer regarding polymer skeletons as inert carriers that need to be shielded but as multifunctional platforms for diversified co-functionalization. Through the targeted integration of complementary interaction modalities, it is feasible to design stationary phases capable of achieving the comprehensive separation of compounds spanning a broad spectrum of polarities and charge properties, thus efficiently addressing the constraints of single-mode materials.

## Figures and Tables

**Table 1:** A comparison of the six common polymerization molding methods.

	Suspension Polymerization	Emulsion Polymerization	Soap-Free Emulsion Polymerization	Precipitation Polymerization	Dispersion Polymerization	Seed Polymerization
Reaction Medium	Water	Water	Water	Organic solvent or organic solvent/water mixture system	Organic solvent or organic solvent/water mixture system	Water or mixed solvent
Polymerization Mode	Homogeneous or heterogeneous	Heterogeneous (multiphase)	Heterogeneous (multiphase)	Heterogeneous	Initially homogeneous, then heterogeneous	Heterogeneous (biphase)
Dispersant	Yes	No	No	No	Yes	Yes
Emulsifier	No	Yes	Trace or no	No	No	Partially yes
Particle Size Distribution	Wide	Narrow	Narrow	Relatively narrow	Narrow	Narrow
Advantages	Low cost, safe, easy to separate	Fast rate, eco-friendly	High product purity, feasible for structural design	High product purity	Simple process, wide monomer applicability	Large product particle size, low cost
Disadvantages	Polydisperse product, impure product	Too small product particle size, impure product	Too small product particle size	High solvent toxicity, low yield	Cannot synthesize high-cross-linking microspheres	High technical requirements, complicated operation

**Table 2:** Common types of emulsifiers.

Type	Examples
Anionic Emulsifiers	Sodium Fatty Acid, Sodium Dodecyl Sulfate, Sodium Dodecyl Sulfonate, Sodium Dodecyl Benzene Sulfonate
Cationic Emulsifiers	Cetyltrimethylammonium Bromide, Dodecyltrimethylammonium Chloride
Zwitterionic Emulsifiers	Amino Acid-Type
Nonionic Emulsifiers	Ethylene Oxide Polymer (Polyoxyethylene-Type), Polyvinyl Alcohol

**Table 3:** The difference between emulsion polymerization and soap-free emulsion polymerization.

Characteristics	Conventional Emulsion Polymerization	Soap-Free Emulsion Polymerization
Essential Relationship	Parent category, basic method	Subcategory, improved/specialized method
Stabilization System	Relies on externally added small-molecule emulsifiers (e.g., SDS); emulsifiers form micelles, which serve as the main polymerization sites	No conventional emulsifiers are added; stabilization is achieved using initiator fragments, hydrophilic comonomers, or ionic monomers themselves
Nucleation Mechanism	Mainly micellar nucleation	Mainly homogeneous nucleation or oligomer micellar nucleation
Latex Particle Characteristics	Broad particle size distribution; particle size adjustable by emulsifier dosage; high solid content (40–60%)	Typically monodisperse, large-sized (usually sub-μm scale) latex particles with clean surfaces; low solid content
Product Purity	Residual emulsifiers in the final polymer (hard to completely remove) may affect product performance (transparency, water resistance, adhesion)	“Clean” polymer latex particle surfaces (no small-molecule emulsifiers); high purity and better performance
Advantages	Mature technology; fast polymerization rate; high molecular weight; low system viscosity; easy heat dissipation; feasible for high-solid-content products	Excellent particle monodispersity; clear surface functional groups; high purity; better biocompatibility; more eco-friendly (reduces small-molecule chemicals)
Disadvantages	Residual emulsifiers impair performance and are hard to eliminate completely	Low solid content; relatively poor polymerization stability; stricter condition control requirements

**Table 4:** The conditions of microspheres obtained using different preparation methods.

Matrix	Preparation Method	APS (μm)	SSA (m^2^/g)	Reference
DVB-TMPT	Microporous Membrane Emulsification	50–60	/	[37]
PMMA	Microporous Membrane Emulsification	0.25–1.60	/	[34]
PVC, PLA, PS	Droplet Microfluidic Technology	50–200	/	[45]
PLGA-PEG, PLGA	Droplet Microfluidic Technology	25.63, 27.89	/	[47]
PS-DVB	Suspension Polymerization	50–500	652	[56]
DVB	Precipitation Polymerization	2.3–4.0	/	[71]
GMA-DVB	Precipitation Polymerization	5.125	434.4	[72]
PS-QDMBD	Dispersion Polymerization	0.6–1.5	/	[80]
GMA-DMB	Seed Polymerization	8.20–11.61	353	[96]
GMA-DVB	Seed Polymerization	6.0	358, 371, 393	[98]
PS-DVB	Seed Polymerization	4.3	338.21	[99]
PS-DVB	Seed Polymerization	10	/	[101]
HPMA-Cl-EGDMA	Seed Polymerization	5	21.73	[102]
PS-DVB	Seed Polymerization	7	68.51	[103]
GMA-DVB	Seed Polymerization	5	353.06–379.86	[104]
PS-DVB	Seed Polymerization	5	78.34	[105]
PS-GMA	Seed Polymerization	6	110.72	[106]
PS-DVB	Seed Polymerization	6.6–7.2	76.143	[107]
EVB-DVB	Seed Polymerization	5.18	37.70	[21]
EVB-DVB	Seed Polymerization	5	625	[20]
EVB-DVB	Seed Polymerization	4.6	45	[108]

**Table 5:** The application of PS-DVB microspheres after chloromethylation and amination as stationary phases in IC.

Functional Group Structure	Chloromethylation Reagent	Amination Reagent	Analyte	Analysis Time ^1^	Reference
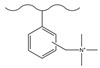	Aminomethyl formic acid/zinc oxide and tin tetrachloride	TMA	F^−^, Cl^−^, NO_2_^−^, HPO_4_^2−^, SO_4_^2−^	30 min	[127]
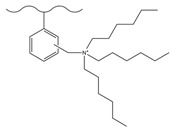	Hydrochloric acid/polyformaldehyde/glacial acetic acid	TMA	F^−^, Cl^−^, Br^−^, NO_3_^−^, ClO_3_^−^, CrO_4_^−^, SO_4_^2−^, S_2_O_3_^2−^	15 min	[128]
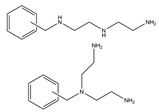	Glacial acetic acid/concentrated hydrochloric acid/formaldehyde	Diethylenetriamine chloride	F^−^, Cl^−^, Br^−^, NO_3_^−^, I^−^, SO_4_^2−^, MoO_4_^2−^, CrO_4_^−^	24 min	[112]
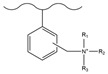	Dimethylmethane/sulfonyl chloride, chlorosulfonic acid	Dimethylaminoethanol	F^−^, Cl^−^, NO_2_^−^, Br^−^, NO_3_^−^, HPO_4_^2−^, SO_4_^2−^, BrO_3_^−^, ClO_2_^−^, ClO_3_^−^	33 min	[129]
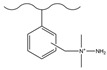	Chlorosulfonic acid/glacial acetic acid/thionyl chloride	N,N-dimethylamine	F^−^, Cl^−^, NO_3_^−^, HPO_4_^2−^, SO_4_^2−^	13 min	[114]
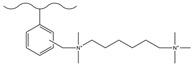	Trioxane/trimethylchlorosilane/chloroform/tin tetrachloride	N,N,N′,N′-tetramethyl-1,6-hexanediamine	*myo*-Inositol, Erythritol, Arabitol, Mannitol, Fucose, Arabinose, Glucose, Sorbose, Ribose, Lactose, Altrose, Raffinose, Maltose	47 min	[130]
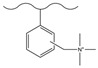	Hydrochloric acid/polyformaldehyde/glacial acetic acid	TMA	HCOO^−^, Cl^−^, Br^−^, NO_3_^−^, ClO_3_^−^	15 min	[131]
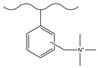	Hydrochloric acid/polyformaldehyde/glacial acetic acid	TMA	N_3_^−^, HOCH_2_COO^−^, HCOO^−^, F^−^, Cl^−^, NO_3_^−^	12 min	[132]
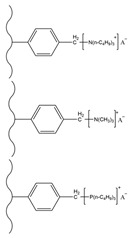	Hydrochloric acid/paraformaldehyde trimethylamine/tributylamine/tributylphosphine	TMA/tributylamine/tributylphosphine	Cl^−^, NO_2_^−^, Br^−^, SO_4_^2−^, NO_3_^−^, S_2_O_3_^2−^	8 min	[133]

^1^ The “analysis time” data in this table and following tables are directly quoted from the original publications. Due to variations in the chromatographic conditions (e.g., column dimensions, flow rate, eluent program) used in each study, the values presented here serve solely as background references for the throughput of the methods reported in each paper. They are not recommended for direct use in cross-study performance comparisons.

**Table 6:** The application of PS-DVB microspheres after Friedel–Crafts alkylation as stationary phase in IC.

Functional Group Structure	Friedel–Crafts Alkylation Reagent	Amination Reagent	Analyte	Analysis Time	Reference
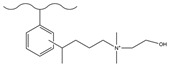	5-bromo-1-pentene/trifluoromethanesulfonic acid	DEMA	F^−^, Cl^−^, NO_2_^−^, Br^−^, NO_3_^−^, HPO_4_^2−^, SO_4_^2−^	35 min	[134,135]
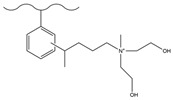	5-bromo-1-pentene	N-methyldiethanolamine	Cl^−^, NO_3_^−^, HPO_4_^2−^, SO_4_^2−^	8 min	[136]

**Table 7:** The application of PS-DVB microspheres after Friedel–Crafts acylation as stationary phase in IC.

Functional Group Structure	Friedel–Crafts Acylation	Reductive Amination Reagent	Alkylation Reagent	Analyte	Analysis Time	Reference
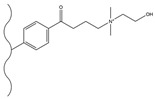	Dichloromethane, aluminum chloride, 4-chlorobutyryl chloride, 4-bromobutyryl chloride	DEMA	/	F^−^, Cl^−^, NO_2_^−^, SO_4_^2−^, Br^−^, NO_3_^−^, HPO_4_^2−^	14 min	[135]
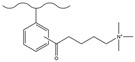	Dichloromethane, aluminum chloride, 3-chloropropionyl chloride, 4-chlorobutyryl chloride, 5-chlorobutyryl chloride	TMA	/	F^−^, Cl^−^, NO_3_^−^, HPO_4_^2−^, SO_4_^2−^	45 min	[137]
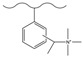	Carbon disulfide, aluminum chloride, acetic anhydride	DMA, sodium cyanoboroh-ydride	Methyl iodide	F^−^, Cl^−^, HPO_4_^2−^, SO_4_^2−^, Br^−^, NO_3_^−^	34 min	[138]
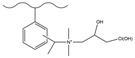	Carbon disulfide, aluminum chloride, acetic anhydride	DMA, sodium cyanoboroh-ydride	Epichlorohyd-rin	F^−^, Cl^−^, NO_2_^−^, Br^−^, NO_3_^−^, HPO_4_^2−^, SO_4_^2−^	30 min	[138]
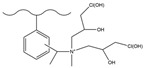	Carbon disulfide, aluminum chloride, acetic anhydride	MA, sodium cyanoboroh-ydride	Epichlorohyd-rin	F^−^, Cl^−^, NO_2_^−^, Br^−^, NO_3_^−^, SO_4_^2−^	8 min	[138]
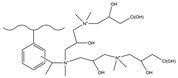	Acetic anhydride	MA, DMA	Epichlorohyd-rin	F^−^, HCOO^−^, Cl^−^, NO_2_^−^, Br^−^, NO_3_^−^, HPO_4_^2−^, SO_4_^2−^	34 min	[139]
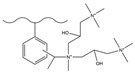	Acetic anhydride, aluminum chloride	MA, sodium cyanoboroh-ydride	GTMA, (3-chloro-2-hydroxyprop-yl) trimethyl CTMA	F^−^, Cl^−^, NO_2_^−^, Br^−^, NO_3_^−^	8 min	[140]
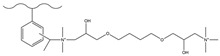	Acetic anhydride, aluminum chloride	DMA, sodium cyanoboroh-ydride	1,4-BDDGE TMA	F^−^, Cl^−^, NO_2_^−^, Br^−^, NO_3_^−^	50 min	[140]
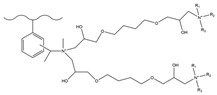	Acetic anhydride, aluminum chloride	MA 1,4-BDDGE	TMA	F^−^, HCOO^−^, Cl^−^, NO_2_^−^, Br^−^, NO_3_^−^, SO_4_^2−^	8 min	[140,141]
			DMEA	F^−^, HCOO^−^, Cl^−^, NO_2_^−^, Br^−^, NO_3_^−^, SO_4_^2−^	12 min	
			MDEA	F^−^, Cl^−^, NO_2_^−^, Br^−^, NO_3_^−^, SO_4_^2−^	11 min	
			TEA	F^−^, HCOO^−^, Cl^−^, NO_2_^−^, Br^−^, NO_3_^−^, SO_4_^2−^	11 min	
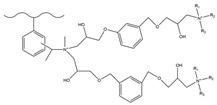	Acetic anhydride, aluminum chloride	MA RDGE	TMA	F^−^, HCOO^−^, Cl^−^, NO_2_^−^, Br^−^, NO_3_^−^, SO_4_^2−^	17 min	[140,141]
			DMEA			
			MDEA			
			TEA			

**Table 8:** The application of PS-DVB microspheres after Friedel–Crafts nitration as stationary phase in IC.

Functional Group Structure	Nitration	The Generation of Amino Groups	Introduced Quaternary Ammonium Salt Groups	Analyte	Analysis Time	Reference
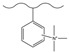	Nitric acid, sulfuric acid	Tin dichloride, hydrochloric acid	Methyl iodide	F^−^, Cl^−^, HPO_4_^2−^, SO_4_^2−^	13 min	[143]
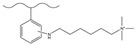	Nitric acid, sulfuric acid	Tin dichloride, hydrochloric acid	1,6-dibromohexane, TMA	F^−^, Cl^−^, Br^−^, HPO_4_^2−^, SO_4_^2−^	31 min	[143]
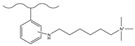	Nitric acid, sulfuric acid	Granular metallic tin, hydrochloric acid	1,2,2,6,6-pentamethylpiperidine	Glucose, Turanose, Maltose, Panose, Maltotriose	9 min	[142]

**Table 9:** The difference between chemical derivatization and surface grafting modification.

	Chemical Derivatization Method	Surface Grafting Method
Action Level & Essence	Atomic/functional group level: Modify atoms or functional groups on the matrix backbone directly via small-molecule organic reactions (e.g., sulfonation, chloromethylation).	Polymer chain/nanostructure level: Connect pre-synthesized or in situ grown polymer chains/functional macromolecules to the matrix surface via covalent bonds.
Functional Layer Structure	No independent “layer” concept: Functional groups are directly bonded to the matrix backbone, serving as an integral part of the matrix.	Clear “functional layer”: An independent, thickness-controllable polymer brush, dendritic macromolecule or nano-coating is formed on the matrix surface.
Impact on Matrix Bulk	Deep impact: Vigorous chemical reactions (strong acids, strong oxidants) may damage the original cross-linking structure, pore size distribution and mechanical strength of the matrix.	Mild impact: Reactions are usually gentle and occur only on the surface, causing little damage to the matrix bulk structure (pores, cross-links).
Controllability & Precision	Low: Reaction sites (e.g., benzene rings of PS-DVB) are randomly distributed; precise control of functional group density, position and chain length is difficult, and batch reproducibility is challenging.	High: Active/controllable polymerization (e.g., ATRP) can be used to precisely regulate the length, density, composition and structure of graft chains, enabling molecular-level designability.
Design Philosophy & Trend	“Terminal” modification: One-step reaction permanently alters the matrix chemistry, making iteration or multifunctionalization difficult. It is a classic but gradually replaced strategy.	“Platform-based” construction: First build an active platform (e.g., initiator layer) on the surface; then, “grow” the functional layer on it. It facilitates multifunctional, multi-level, iterative precision design, representing the modern mainstream and frontier direction.

**Table 10:** The separation of anion chromatography stationary phases through different functionalization methods.

Matrix	Functionalization Method	Analyte	Analysis Time	Reference
PS-DVB	Surface grafting	F^−^, Cl^−^, NO_2_^−^, Br^−^, NO_3_^−^, SO_4_^2−^	15 min	[186]
PS-DVB	Surface grafting	F^−^, Cl^−^, NO_2_^−^, Br^−^, SO_4_^2−^, NO_3_^−^, HPO_4_^2−^	12 min	[188]
PS-DVB	Surface grafting	F^−^, Cl^−^, NO_3_^−^, PO_4_^3−^, SO_4_^2−^, I^−^, SCN^−^, S_2_O_3_^2−^	11 min	[124]
GMA-DVB	Surface grafting	F^−^, Cl^−^, NO_2_^−^, Br^−^, NO_3_^−^, SO_4_^2−^, HPO_4_^2−^	16 min	[172]
PS-DVB	Latex agglomeration	F^−^, Cl^−^, NO_2_^−^, Br^−^, NO_3_^−^, PO_4_^3−^, SO_4_^2−^	8 min	[181]
PS-DVB	Latex agglomeration	F^−^, Cl^−^, ClO_2_^−^, NO_2_^−^, BrO_3_^−^, Br^−^, NO_3_^−^, ClO_3_^−^	12 min	[189]
PS-DVB	Hyperbranching	F^−^, HCOO^−^, Cl^−^, BrO_3_^−^, NO_2_^−^, Br^−^, SO_4_^2−^, NO_3_^−^	15 min	[120]
PS-DVB	Hyperbranching	F^−^, Cl^−^, NO_2_^−^, Br^−^, NO_3_^−^, SO_4_^2−^, PO_4_^3−^	7 min	[123]
EVB-DVB	Hyperbranching	F^−^, Cl^−^, Br^−^, NO_2_^−^, ClO_2_^−^, BrO_3_^−^, ClO_3_^−^, NO_3_^−^	13 min	[21]
EVB-DVB	Hyperbranching	F^−^, HCOO^−^, Cl^−^, NO_2_^−^, SO_4_^2−^, Br^−^, NO_3_^−^, ClO_3_^−^, PO_4_^3−^	17 min	[183]

## Data Availability

No new data were created or analyzed in this study. Data sharing is not applicable to this article.

## Abbreviations

The following abbreviations are used in this manuscript:

IC	Ion Chromatography
COF	Covalent Organic Framework
MOF	Metal–Organic Framework
PS-DVB	Polystyrene–Divinylbenzene
EVB-DVB	Ethylvinylbenzene–Divinylbenzene
SSA	Specific Surface Area
GMA	Glycidyl Methacrylate
O/W	Oil-in-Water
W/O	Water-in-Oil
PSD	Particle Size Distribution
APS	Average Particle Size
CV	Coefficient of Variation
PMMA	Poly(Methyl Methacrylate)
PVA	Poly(Vinyl Alcohol)
PVC	Polyvinyl Chloride
PLA	Polylactic Acid
PVDF-HFP	Polyvinylidene Fluoride–Hexafluoropropylene
PLGA	Poly(Lactic-Co-Glycolic Acid)
HOPMs	Highly Open Porous Microspheres
PLGA-PEG	Poly(Lactic-Co-Glycolic Acid)-Polyethylene Glycol
MW	Molecular Weight
ATRP	Atom Transfer Radical Polymerization
RAFT	Reversible Addition–Fragmentation Chain Transfer
PAMAM	Polyamidoamine
AGE	Allyl Glycidyl Ether
PEI	Polyethyleneimine
BDDGE	1,4-Butanediol Diglycidyl Ether
MA	Methylamine
DMA	Dimethylamine
TMA	Trimethylamine
DMEA	Dimethylethanolamine
MDEA	Methyldiethanolamine
TEA	Triethanolamine
GTMA	Glycidyltrimethylammonium Chloride
CTMA	(3-Chloro-2-Hydroxypropyl)Trimethylammonium Chloride
AIBN	Azobisisobutyronitrile
BPO	Benzoyl Peroxide
PVP	Polyvinylpyrrolidone
CTAB	Cetyltrimethylammonium Bromide
SDS	Sodium Dodecyl Sulfate
NaSS	Sodium p-Styrenesulfonate
QDMDB	N′-Dimethyl-N-n-Dodecyl-N-2-Methacryloyloxyethyl Ammonium Bromide
HPMA-Cl	3-Chloro-2-Hydroxypropyl Methacrylate
EGDMA	Ethylene Glycol Dimethacrylate
RSD	Relative Standard Deviations
cdG	Carbon-Doped Graphene
HCSs	Hydrothermal Carbonaceous Spheres
PINN	Physics-Informed Neural Network
